# Benchmarking HLA genotyping and clarifying HLA impact on survival in tumor immunotherapy

**DOI:** 10.1002/1878-0261.12895

**Published:** 2021-01-24

**Authors:** Xiangyong Li, Chi Zhou, Ke Chen, Bingding Huang, Qi Liu, Hao Ye

**Affiliations:** ^1^ Key Laboratory of Spine and Spinal Cord Injury Repair and Regeneration (Tongji University) Ministry of Education Orthopaedic Department of Tongji Hospital Bioinformatics Department School of Life Sciences and Technology Tongji University Shanghai China; ^2^ Sinotech Genomics Shenzhen China; ^3^ College of Big Data and Internet Shenzhen Technology University Shenzhen China; ^4^ CStone Pharmaceuticals Co., Ltd. Suzhou China

**Keywords:** HLA genotyping, immune cytolytic activity, tumor immunotherapy, tumor mutation burden

## Abstract

Human leukocyte antigen (HLA) genotyping gains intensive attention due to its critical role in cancer immunotherapy. It is still a challenging issue to generate reliable HLA genotyping results through *in silico* tools. In addition, the survival impact of HLA alleles in tumor prognosis and immunotherapy remains controversial. In this study, the benchmarking of HLA genotyping on TCGA is performed and a ‘Gun‐Bullet’ model which helps to clarify the survival impact of HLA allele is presented. The performance of HLA class I genotyping is generally better than class II. POLYSOLVER, OptiType, and xHLA perform generally better at HLA class I calling with an accuracy of 0.954, 0.949, and 0.937, respectively. HLA‐HD obtained the highest accuracy of 0.904 on HLA class II alleles calling. Each HLA genotyping tool displayed specific error patterns. The ensemble HLA calling from the top‐3 tools is superior to any individual one. HLA alleles show distinct survival impact among cancers. Cytolytic activity (CYT) was proposed as the underlying mechanism to interpret the survival impact of HLA alleles in the ‘Gun‐Bullet’ model for fighting cancer. A strong HLA allele plus a high tumor mutation burden (TMB) could stimulate intensive immune CYT, leading to extended survival. We established an up to now most reliable TCGA HLA benchmark dataset, composing of concordance alleles generated from eight prevalently used HLA genotyping tools. Our findings indicate that reliable HLA genotyping should be performed based on concordance alleles integrating multiple tools and incorporating TMB background with HLA genotype, which helps to improve the survival prediction compared to HLA genotyping alone.

## Graphical abstract

Human leucocyte antigen (HLA) genotyping can provide important insights for tumor prognosis and cancer immunotherapy. We performed comprehensive HLA benchmarking in TCGA cohorts, based on allele concordance results generated from eight *in silico* tools. Moreover, we investigated the impact of HLA alleles on tumor survival. Our proposed ‘Gun‐Bullet’ model considers immune cytotoxicity to interpret the survival impact of HLA alleles and tumor mutation burden.

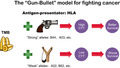

**Abbreviations**
BRCA: breast invasive carcinoma
CYT: cytolytic activity
DCB: durable clinical benefit
HLA: human leukocyte antigen
ICB: immune checkpoint blocker
LIHC: liver hepatocellular carcinoma
LOH: loss of heterozygous
NDB: no durable benefit
NGS: next‐generation sequencing
NK: natural killer
NSCLC: nonsmall‐cell lung cancer
OS: overall survival
OV: ovarian serous cystadenocarcinoma
PBMCs: peripheral blood mononuclear cells
READ: rectum adenocarcinoma
SKCM: skin cutaneous melanoma
TMB: tumor mutation burden
WES: whole exome sequencing

## 1 Introduction

Human leukocyte antigen (HLA) plays a critical role in antigen presentation during self or nonself immune recognition process, that ultimately launch the initial immune signaling response. It locates at the most polymorphic region in chromosome 6p21. 19 970 individual HLA proteins encoded by 28 786 alleles were reported in latest IMGT/HLA database [1] (Release 3.42.0). Typically, HLA system contains two copies of HLA‐A, B, C, DRB1, DQB1, DPB1, DQA1, etc. Such combination of different HLA alleles makes the dramatically distinct immune response between individuals, even under the same internal/external stimulation. Therefore, accurate HLA genotyping and stringent matching of patient and donor are extremely important in organ transplantation. Any slight mismatches may lead to a serious graft‐versus‐host rejection. Currently, capillary electrophoresis‐based dye‐terminator Sanger sequencing is served as the gold‐standard approach for high resolution HLA genotyping in clinical laboratories. Since two copies of alleles are sequenced together in one reaction, it is difficult to determine whether the detected SNPs come from the same chromosome (*cis*) or from opposite chromosome (*trans*). Several rounds of additional sequencing are needed to identify *cis/trans* polymorphism. In addition, the increasing quantity of new HLA alleles would introduce more laborious efforts on primer designing and sequencing. Due to such limitations, the concordance rate of Sanger sequencing‐based HLA genotyping is about 84% among different laboratories [2]. Obviously, the traditional labor‐intensive and accuracy‐low methods are far behind clinical requirement.

The development of next‐generation sequencing (NGS) technology has changed the landscape of HLA genotyping. Since the DNA fragments are independently amplified and sequenced, it could dramatically reduce the ambiguities of *cis/trans* polymorphism occurs in Sanger sequencing. Generally, three typical processes are implemented in NGS‐based HLA genotyping: (a) reads alignment toward known HLA reference sequences, such as IMGT/HLA sequence database; (b) candidate HLA alleles identification through assembling the aligned reads into contigs; and (c) HLA allele pair inference by scoring the coverage balance of any two identified candidate alleles. Up to now, dozens of bioinformatics tools have been developed for NGS‐based HLA genotyping. All the tools were smartly designed to optimize the accuracy and speed on the mentioned three processes. For example, POLYSOLVER [3] adopted a two‐step Bayesian classification model to infer HLA allele pair, with careful consideration of sequence quality, insert sizes, and ethnicity‐dependent allele prior probability. OptiType [4] constructed a binary hit matrix indicating the alleles that a specific reads sequence could be aligned with minimum mismatches. Integer linear programming is subsequently used to maximum the number of explainable reads by selecting one or two alleles. A recently developed tool called Kourami [5] introduced a graph‐guided assembly strategy to infer the most likely HLA allele pair. Since the gene level partial order graphs were prebuilt from known HLA sequences and remodified by reads alignment, it confers the ability of discovering novel alleles that do not appear in the database. Most of these HLA genotyping tools displayed the best performance in their releasing demo dataset, an unbiased and comprehensive benchmarking with large scale curated benchmark datasets on these tools, however, is still lacking in this field.

In addition, due to the essential role in the molecular mechanism of immune recognition, HLA gains a lot of scientific interests on immune oncology in recent years. Rachel [6, 7] found that HLA allele provide a tumor evolutionary pressure through restricting the oncogenic mutational landscape in TCGA cohort. The frequent driver mutations are universally poorly presented by HLA, since the tumor clones carrying the strongly presented mutations had already been killed by immune system. HLA allele‐specific loss of heterozygous (LOH) was verified as an important immune evasive mechanism of nonsmall‐cell lung cancer (NSCLC), which could impair the ability of recognizing tumor antigens in immune system [8]. Furthermore, HLA may serve as an independent biomarker for immune checkpoint blocker (ICB) therapy. Positive correlation between diversity of HLA class I alleles and clinical benefit was found by Chowell [9] in ICB‐treated melanoma cohort. Since a broader antigen profile would be presented in heterozygous HLA allele than homozygous, the likelihood of benefit would be subsequently increased. What's more, the presence of HLA class I supertype B44 was correlated with a longer overall survival (OS). However, it remains controversial on the prognostic effect of HLA under ICB therapy. In a recently released study, Negrao *et al*. [10] found HLA supertype is not correlated with OS in ICB‐treated advanced NSCLC cohort collected in MD Anderson Cancer Center. Therefore, it is worthy to investigate why such conflict occurs in these studies.

Cytolytic activity (CYT) is the ultimate effect of HLA‐antigen stimulated immune response, which initially quantified by Rooney [11] as the geometric mean expression of GZMA and PRF1. Theoretically, neoantigen is likely to drive CYT. It has been partially verified through the positive correlation between neoantigen load and CYT across multiple tumor types in TCGA. Moreover, a high CYT is associated with a modest but significant pan‐cancer survival benefit. Therefore, it is worthy to investigate that whether CYT could bridge the HLA‐antigen initialized immune response and the OS.

Taking together, an objective benchmark of available HLA genotyping tools as well as a systematic investigation of the survival impact of HLA genotype based on the well curated TCGA data and clinical cohorts treated with ICBs are needed. We investigated these two problems in one study since the accurate HLA calling and the following analysis of their survival impact are fundamental for immunotherapy. In this study, we performed a comprehensive HLA benchmark in TCGA cohorts, composing of concordance results generated from eight frequently used or latest developed tools, including POLYSOLVER, OptiType, xHLA, HLA‐HD, hla‐genotyper, SOAP‐HLA, HLA‐VBSeq, and Kourami. In addition, the influence of HLA allele on immune CYT and survival is further investigated. Moreover, a ‘Gun‐Bullet’ model was proposed to interpret the survival impact of HLA alleles through CYT. Specifically, the relationship of HLA and tumor mutation burden (TMB) was similar to ‘Gun’ and ‘Bullet’. The integrative effect of HLA allele and TMB was likely to induce CYT and subsequently influence survival. We set out to address several following questions, which is closely related to the clarifying of the controversial issue of survival impact of HLA allele in immunotherapy, including: (a) systematically investigation and comparison of the performance of individual HLA genotyping tools and the ensemble one; (b) what is the frequency error pattern in each HLA genotyping tool? and (c) what is the underlying explanation to address the controversial issue on the prognostic effect of HLA allele in survival and tumor immunotherapy?

## 2 Materials and methods

### 2.1 Study design

**Fig. 1:**
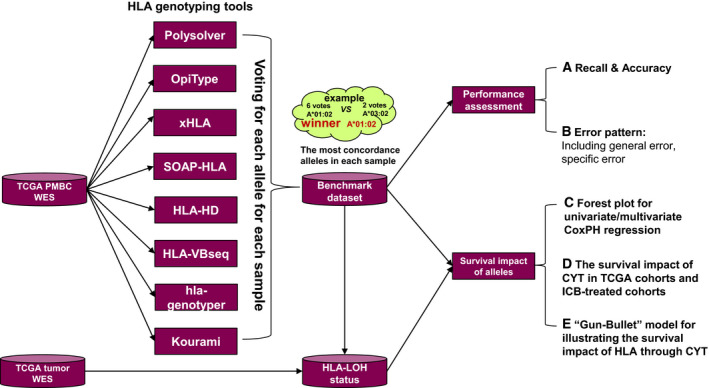
The workflow of study design.

As shown in Fig. 1, the benchmark dataset was composed of the most concordance HLA alleles generated by eight prevalently used HLA genotyping tools based on peripheral blood mononuclear cells (PBMCs) WES data in TCGA. In addition, HLA allele‐specific LOH were called from tumor WES data in TCGA and the benchmark dataset. Then, the HLA genotyping performance of each tools was evaluated, including recall, accuracy, and error patterns. What's more, the survival impact of HLA alleles was also investigated on TCGA cohorts. Specifically, univariate and multivariate CoxPH regression were used to detect the HLA alleles that significantly influencing the OS of patient. Since the survival benefit of CYT had been proved in TCGA pan‐cancer cohorts in Rooney's study [11], we further investigated the survival impact of CYT on independent cohorts under ICB treatment. Finally, a ‘Gun‐Bullet’ model was proposed to interpret the survival impact of HLA based on CYT. The study methodologies conformed to the standards set by the Declaration of Helsinki. Detailed clinical information regarding the cohorts collected in the study can be found at Table S1.

### 2.2 WES data processing

A total of 10 479 tumor‐normal paired whole exome sequencing (WES) data (PBMCs: sample code ‘10’; tumor: sample code ‘01’) were retrieved from TCGA website. We exclusively downloaded the reads aligned on HLA region, following the GDC API guidance to perform BAM slicing at hg38 genome loci: chr6:28032222‐34032223. Then, the WES‐derived germline HLA sequence data were imported for HLA genotyping with eight prevalently used tools. Specially, HLA class I calling is exclusively available for POLYSOLVER [3] and OptiType [4]. HLA‐HD [12], hla‐genotyper (https://pypi.org/project/hla‐genotyper/), SOAP‐HLA (http://soap.genomics.org.cn/SOAP‐HLA.html.), xHLA [13], HLA‐VBSeq [14], and Kourami [15] were used for both HLA Class I and HLA Class II genotyping. All the HLA alleles generated by the eight tools were normalized into 4‐digit resolution. For example, A01:01:03 was transformed to A01:01.

### 2.3 TCGA benchmark dataset of HLA class I and class II

As no golden standard dataset is available for HLA genotyping, it is critical to create an HLA benchmark dataset representing the benchmark in silico. Herein, we take advantages of the basic idea of ensemble learning by equally treat each HLA genotyping tool as an expert. Then, each copy of HLA gene including A, B, C, DRB1, DPB1, DQA1, and DQB1 was, respectively, voted by these ‘experts’ with an ensemble way. The most concordance allele would obtain the highest votes, and they are selected as the ‘ground truth’ in the benchmark analysis. In order to avoid the ambiguous calling, only the most concordance allele with at least two votes would be curated into the benchmark dataset. Then, each sample was assigned a group of HLA genes that composed of the most concordance alleles with highest votes. Twelve HLA supertypes were also conferred to TCGA samples, strictly following the HLA class I allele classification approach mentioned in Chowell's study [9] and Sidney' study [16]. It should be noted that the benchmark dataset derived from such highest concordance HLA alleles are not exactly equal to the ground truth; however, the ambiguous calling alleles against such benchmark dataset would nevertheless indicate the risk of genotyping the specific allele in that tool. The HLA alleles with high error rate genotyped by single tool would be taken with caution.

### 2.4 HLA‐LOH status assessment

The HLA allele‐specific LOH calling was implemented by LOHHLA [8] (https://bitbucket.org/mcgranahanlab/lohhla/src/master/). Since only allele sequence at genome level is supported by LOHHLA program, the consensus 4‐digit protein level HLA allele generated above was transformed to higher resolution allele with longest genome sequence. For example, A01:09:01:02 with 3359bp in genome length was used for LOH assessment on 4‐digit allele A01:09. Allele‐specific LOH event was detected, according to the allelic coverage imbalance status (*P* value ≤ 0.05).

### 2.5 Evaluation metrics for HLA genotyping benchmark

HLA class I and class II genotyping performance were separately evaluated, since not all tools could detect two HLA classes. Recall and accuracy defined by Thorne's study [2] were used for performance evaluation. These two metrics were calculated as the following formula:

(1) $$\text{Recall}=\frac{\text{number}\;\text{of}\;\text{right}\;\text{alleles}}{\text{number}\;\text{of}\;\text{right}\;\text{alleles}+\text{number}\;\text{of}\;\text{wrong}\;\text{allele}}$$

(2) $$\text{Accuracy}=\frac{\text{number}\;\text{of}\;\text{right}\;\text{alleles}}{\text{number}\;\text{of}\;\text{right}\;\text{alleles}+\text{number}\;\text{of}\;\text{wrong}\;\text{alleles}+\text{number}\;\text{of}\;\text{uncalled}\;\text{alleles}}$$

Taking HLA class I alleles as an example, two copy of HLA‐A, B, and C alleles predicted by eight tools were compared with the corresponding benchmark dataset for each patient, respectively. Then, the predicted alleles could be classified into three types. Right allele indicates the allele is covered by the benchmark dataset, while the wrong allele means the allele is absent in the benchmark dataset. The alleles that failed for genotyping by the tool were defined as uncalled alleles.

### 2.6 HLA genotyping error pattern in each tool

In order to investigate the error calling bias in each tool, general error rate and specific error rate were calculated through formula (3) and (4). The high general error rate of the allele indicates a high probability of making mistakes by the tool. Specific error rate could quantify the preference of ambiguous calling on allele pairs. For example, 435 A25:01 allelic counts were detected in TCGA HLA benchmark dataset. Three hundred and seventy‐one of them were miscalled as other alleles by POLYSOLVER. Specifically, 343 cases were incorrectly called as A26:01. Then, the general error rate on A25:01 calling in POLYSOLVER is 85.29% (371/435), and the specific error rate on the allele pair A25:01‐A26:01 is 92.45% (343/371). Herein, allelic counts were utilized in general error rate and specific error rate calculation. It is different from population counts. If a patient carried heterozygous HLA‐A alleles A25:01/A26:01, both the allelic counts and population counts of A25:01 are equal to 1. If A25:01 is present as homozygous in that patient, the allele counts of A25:01 are equal to 2, while the population counts of A25:01 are still equal to 1.

(3) $$\text{general}\;\text{error}\;\text{rate}=\frac{\text{counts}\;\text{of}\;\text{incorrect}\;\text{genotyping}\;\text{on}\;\text{allele}\;i}{\text{counts}\;\text{of}\;\text{allele}\;i\;\text{in}\;\text{benchmark}\;\text{data}}$$

(4) $$\text{specific}\;\text{error}\;\text{rate}=\frac{\text{counts}\;\text{of}\;\text{allele}\;i\;\text{that}\;\text{miscalled}\;\text{as}\;\text{allele}\;j}{\text{counts}\;\text{of}\;\text{incorrect}\;\text{genotyping}\;\text{on}\;\text{allele}\;i}$$

### 2.7 Clinical data processing

With browsing the 33 TCGA projects on https://portal.gdc.cancer.gov/projects/, the clinical information table for each project was downloaded by clicking the ‘*Clinical*’ at top right corner of the webpage. OS of patients was generated by the column ‘days_to_death’, ‘days_to_last_follow_up’, and ‘vital_status’ in the tsv file. Specifically, if ‘vital_status’ is filled with alive, the OS of such patient is defined as the number of days in column ‘days_to_last_follow_up’. While if dead is found in column ‘vital_status’, OS is indicated by the value in column ‘days_to_death’. Tumor stages are classified into early and advanced based on the column ‘tumor_stage’. In detail, early stage contains stage 0, i, ii, while stage iii, iv, and x were included in advanced stage.

### 2.8 CYT calculation

Releasing perforin and granzymes into target cell is the major molecular mechanism underlying the destroy ability of immune effector cells [17, 18]. Since perforin and granzymes were exclusively secreted by immune effector cytotoxic T lymphocytes and natural killer (NK) cells, the expression of GZMA and PRF1 gene detected in bulk tumor tissue could be quantified as an indicator of the intratumoral immune CYT [19, 20]. In this study, we retrieved the mRNA expression GZMA and PRF1 among TCGA tumor samples from UCSC Xena website (https://xenabrowser.net/datapages/). Then, the CYT of each sample was defined as the geometric mean of GZMA and PRF1 (as expressed in TPM, 0.01 offset), following Rooney's study [11]. Regarding to each specific cancer condition, the patients were equally separated into two groups (high CYT and low CYT) by the median CYT value under the specific cancer condition.

### 2.9 TMB calculation

The raw somatic mutations results generated by *mutect* in *maf* format were downloaded from TCGA website (https://portal.gdc.cancer.gov/). Then, the nonsynonymous variants located in coding region were processing for TMB calculation [21, 22]. In detail, the column ‘One_Consequence’ contains one of the key words listed below would be identified as nonsynonymous somatic mutations. Such key words pool contains ‘missense_variant’, ‘frameshift_variant’, ‘stop_gained’, ‘inframe_insertion’, ‘splice_region_variant’, ‘splice_donor_variant’, ‘inframe_deletion’, ‘splice_acceptor_variant’, ‘protein_altering_variant’, ‘stop_lost’, ‘start_lost’, ‘stop_retained_variant’, and ‘coding_sequence_variant’. TMB are calculated as the number of nonsynonymous somatic mutations divided by the length of coding region. Regarding to each specific cancer condition, high‐ and low‐TMB groups were equally identified through the comparison with the median TMB value under the specific cancer condition.

### 2.10 Clinical cohort treated with immune checkpoint blockers

Analyses were conducted on five cohorts (including three melanoma cohorts and two NSCLC cohorts) treated with ICBs. The Van Allen cohort [23] consisted of 41 melanoma patients treated with ipilimumab (anti‐CTLA4 therapy) (Table S1A). The Tuba cohort [24] consisted of 73 melanoma patients treated with Nivolumab/Pembrolizumab (anti‐PD‐1 monotherapy) or combined anti‐PD‐1 and anti‐CTLA‐4 immunotherapy. Patient outcomes were classified as responding to therapy (CR or PR, *n* = 39) or not responding to therapy (SD or PD, *n* = 34) (Table S1B). Both RNA‐seq and WES data were available in Van Allen's melanoma cohort [23]. The processing results (including HLA, TMB, CYT) of the two melanoma cohorts were retrieved from our previous studies [25, 26, 27]. Chowell's melanoma cohort [9] contains 164 patients under anti‐CTLA4 treatment. The HLA and TMB information were collected from the supplementary of the original paper. (Table S1C). Jae‐Won Cho's NSCLC cohort [28] contained five patients with durable clinical benefit (DCB) and 11 patients in no durable benefit (NDB) group under anti‐PD1 therapy. Jeong Yeon Kim's NSCLC cohort [29] consisted of eight patients with DCB and 19 patients with NDB under anti‐PD1 treatment. The gene expression profiling results were generated through STAR [30] alignment and subsequent expression quantification in RSEM [31]. (Table S1D,E). OS data utilized in the survival analyses and TMB information were also retrieved from original study. The HLA alleles and LOH of HLA were generated as described in Materials and methods.

### 2.11 Statistical analysis

Survival analysis was implemented by python package *lifelines* (https://github.com/CamDavidsonPilon/lifelines). In detail, hazard ratio was calculated by Cox proportional hazards regression model with the function *CoxPHFitter*. Subsequently, R package *forestplot* was utilized for results visualization. Kaplan–Meier survival plot was generated by function *KaplanMeierFitter* in python package *lifelines*.

## 3 Results

### 3.1 The performance of HLA class I genotyping is generally better than class II

**Fig. 2:**
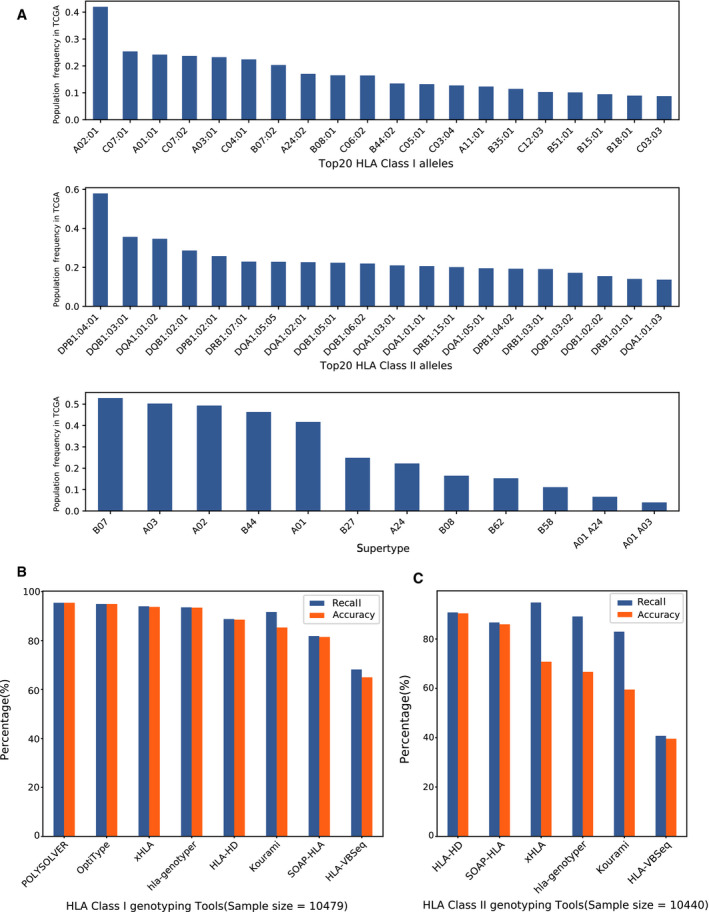
The summary of HLA alleles and genotyping performance in TCGA benchmark dataset. (A). The Top‐20 HLA class I alleles with high population frequency, the Top‐20 HLA class II alleles with high population frequency and the population frequency of 12 HLA supertypes in TCGA benchmark dataset. The population frequency was defined as the percentage of samples carrying a specific allele among TCGA benchmark samples. (B). The recall and accuracy on HLA class I alleles calling. (C) The recall and accuracy on HLA class II alleles calling.

Since no golden standard HLA genotyping results are available in TCGA, we try to computationally curate a comprehensive benchmark data taken as the ground truth with ensemble strategy. In detail, the 4‐digit HLA class I and II benchmark dataset were generated by the concordance analysis on HLA genotyping results of eight tools. Such HLA benchmark dataset contains 10 479 samples with HLA class I results and 10 440 samples with class II results, respectively. Each allele in the benchmark dataset derives from the most concordance alleles that successfully reported by the eight tools. The population frequency of the top 20 HLA class I alleles, class II alleles, and 12 supertypes in TCGA cohort was displayed in Fig. 2A (see the full population frequency list in Table S2). The population frequency across the alleles is quite imbalance. A02:01 is the dominant HLA class I allele in TCGA cohort with the population frequency at 41.99% (allelic frequency is 8.03%), which was detected in 4400 individual patients. Six thousand and forty‐nine patients carrying DPB1:04:01 that occupied 57.94% in TCGA cohort (the corresponding allelic frequency in TCGA is 9.00%). B07, A03, A02, B44, and A01 are top five HLA supertypes with population frequency more than 40%.

We compared the prediction results of each tool against the benchmark dataset. The recall and accuracy of eight tools on HLA class I genotyping were displayed in Fig. 2B. The best performance of HLA class I genotyping was found in POLYSOLVER. Both recall and accuracy are detected above 0.954. 3/10 479 samples were failed for HLA class I genotyping on POLYSOLVER. However, OpiType, xHLA, HLA‐HD, and SOAP‐HLA were successful to generate HLA class I results on all the samples. Specially, OpiType displayed a very good performance with accuracy at 0.949, which is slightly close to POLYSOLVER.

Since the top two HLA class I predictors (POLYSOLVER and OpiType) do not support HLA class II genotyping, the performance of the other six tools was evaluated. Specifically, HLA‐HD, SOAP‐HLA, and HLA‐VBSeq are able to call four major class II genes, including DRB1, DPB1, DQA1, and DQB1. DRB1, DQB1, and DPB1 were supported by xHLA. Kourami, hla‐genotyper, and HLA‐VBSeq were designed for calling DRB1, DQA1, and DQB1. As shown in Fig. 2C, HLA‐HD obtained the highest accuracy at 0.904 on calling DRB1, DPB1, DQA1, and DQB1. The highest recall was achieved by xHLA at 0.948. However, the accuracy of xHLA was dramatically dropped to 0.708, due to its disability on DQA1 calling. If it is specifically focused on the three genes (DRB1, DQB1, and DPB1) supported by xHLA, the corresponding accuracy could increase to 0.944, which indicates that xHLA shows a high performance on calling the supported class II genes.

### 3.2 Frequently miscalled HLA class I alleles in the tested eight tools

**Fig. 3:**
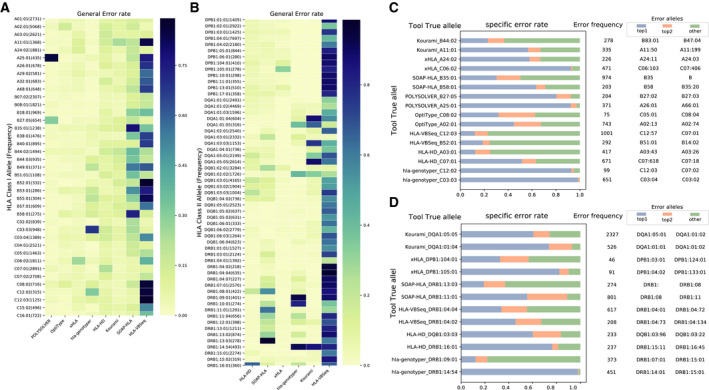
Overview of errors in each tool. (A). Heat map of HLA Class I alleles’ error rate across eight tools. (B). Heat map of HLA Class II alleles’ error rate across six tools. The alleles were picked out according to two criterions: 1). The frequency of the alleles in benchmark dataset ≥200. 2). The general error rate is no less than 0.05 in at least one of the tools. (C). The specific error rate of HLA Class I alleles in eight tools. Each row means a specific error rate pattern made by a tool. The blue bar indicates the percentage of highest specific error alleles. The orange bar means the percentage of top 2 high error alleles. The green bar represents the percentage of the other alleles. For example, in the first row started with “Kourami_B44:02”, the allelic counts of B44:02 mis‐assigned as other alleles by Kourami is 278. Specifically, 64 of them were reported as B83:01 in blue bar (specific error rate 23.02%). 38 of them were identified as B47:04 in orange bar (specific error rate: 13.67%). 176 of them were incorrectly assigned as other alleles in green bar. (D). The specific error rate of HLA Class II alleles in six tools.

In order to investigate the error bias in each tool, we calculated the general error rate of HLA alleles through comparing the HLA calling results with the benchmark dataset. Thirty‐nine frequently miscalled class I alleles with high general error rates were picked out according to two criterions: (a). the allelic frequency in benchmark dataset ≥ 200 and (b) the general error rate is no less than 0.05 in at least one tool. As shown in Fig. 3A and Table S3A (see the full HLA class I error information in Table S4 **)**, each tool revealed distinct error patterns. 85.29% (371/435) of A25:01 in benchmark dataset were miscalled in POLYSOLVER. hla‐genotyper usually makes mistake on C03:03 calling with a general error rate at 68.67% (651/948). C07:01 is the top miscalled allele in HLA‐HD with a general error rate at 23.21% (671/2891). xHLA tend to make mistakes on C06:02 calling with a general rate at 26.01%. The top miscalled alleles in Kourami, SOAP‐HLA, and HLA‐VBSeq are A11:01, B35:01, and C12:03, respectively. However, it seems that no strong error bias was found in OptiType, since the only two alleles (A02:01 and C08:02) were detected with general error rates higher than 10%.

### 3.3 Frequently miscalled HLA class II alleles in the tested six tools

Using the same processing approach mentioned above, 57 HLA class II alleles with high general error rates were displayed in Fig. 3B and Table S3B (see the full HLA class II error information in Table S5). Comparing with HLA Class I calling, the general error rate is high on calling class II alleles in each tool. Specifically, some class II alleles were called with general error rates higher than 90% in several tools. For example, 98.56% DRB1:13:03 in TCGA HLA benchmark dataset were miscalled by SOAP‐HLA. It is challenging for Kourami to identify DQA1:05:05, DQA1:01:04, and DRB1:14:54 with the error rate more than 85%. HLA‐HD tends to make mistake on calling DRB1:16:01 with a general error rate at 65.83%. However, no dominant error alleles in DRB1, DQB1, and DPB1 were observed in xHLA. HLA‐VBSeq incorrectly called a lot of class II alleles with a general error rate higher than 90%, including DRB1:04:02, DRB1:04:04, DRB1:04:07, and DRB1:07:01.

### 3.4 The preference of miscalled HLA allele pairs

With the aim of gaining insights on the ambiguous calling HLA pairs in each tool, we further investigated the error components of top two miscalled alleles in each tool. As shown in Fig. 3C, serval specific error HLA class I allele pairs were indeed existing among the calling results by each individual tool. For example, 92.45% (343/371) errors on A25:01 calling is derived from A26:01 in POLYSOLVER. 436 C06:02 cases miscalling to C06:103 were found in xHLA, which occupied 92.57% errors that occurs in C06:02. hla‐genotyper tends to make mistakes on calling C03:03 as C03:04 and C12:02 as C12:03. Such specific errors occupied more than 93% error cases in the two alleles. Among the 203 miscalled B58:01 alleles, 63.55% (129) of them were assigned as B58 by SOAP‐HLA. 190 A11:01 cases were identified as A11:50, which composed of 56.72% A11:01 miscalling cases made by Kourami. Reporting A02:01 as A02:13 is the highest error observed in OpiType, which occupied 44.82% A02:01 miscalling cases.

The error components of top two HLA class II alleles in each tool were displayed in Fig. 3D. Similar to that in HLA class I allele, several dominant error allele pairs were detected in each tool. For example, DPB1:04:02 is the dominant error allele in DPB1:105:01 calling through xHLA with the specific error rate at 82.42%. 442 DRB1:14:54 cases were called as DBR1:14:01 by hla‐genotyper, which occupied 98.00% DRB1:14:54 miscalling cases.

### 3.5 Integration of HLA calling from the top‐3 tools is superior to individual one

**Fig. 4:**
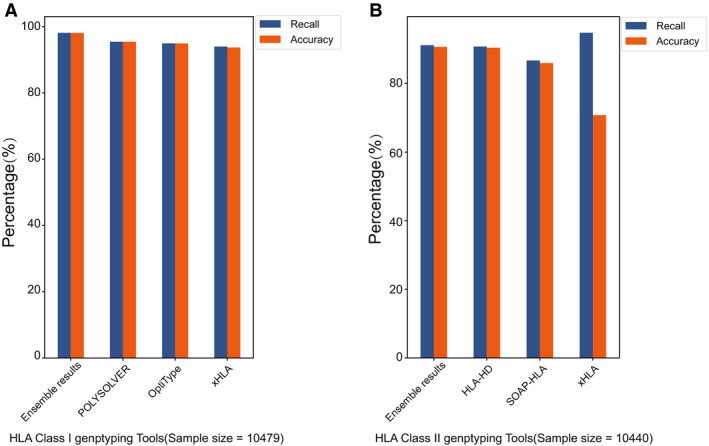
The recall and accuracy of ensemble results in HLA class I and II genotyping. (A). The recall and accuracy on HLA class I calling in POLYSOLVER, OpiType, xHLA and their ensemble results. (B). The recall and accuracy on HLA class II calling in HLA‐HD, SOAP‐HLA, xHLA and their ensemble results.

Generally, none of the tested tools achieved the high accuracy that claimed in their respective papers. Probably such situation is caused by the relatively small benchmark data size. For example, only three trio samples were used in HLA‐VBSeq performance assessment [14]. Kourami was tested on 12 trios samples [15]. However, the large TCGA HLA benchmark dataset covering ~ 10 479 samples curated in our study would ensure a robust and unbiased performance assessment. POLYSOLVER, OpiType, and xHLA ranked as top three HLA class I genotyping tools with the high accuracy ranging from 0.937 to 0.954. However, HLA‐HD, SOAP‐HLA, and xHLA obtain the relatively high performance (accuracy 0.708–0.904) on HLA class II alleles DRB1, DPB1, DQA1, and DQB1 calling. In addition, distinct error patterns were found among the HLA genotyping tools. POLYSOLVER tends to make mistakes on A25:01 calling, but such error is unlikely to happen on OpiType. Therefore, it is unwise to make decision on HLA genotyping based on individual tool. Such distinct error pattern may suggest that the incorrectly called alleles would be efficiently avoided through concordance analysis from multiple HLA genotyping tools. To this end, we try to create an ensemble result from the top three tools following the approach that quite similar to benchmark dataset curation. If the allele is reported by no less than two tools, it will be considered as the true allele. Otherwise, if no overlapping allele is found in the three tools, the allele reported by the tool with a highest accuracy will be assigned as the true allele. Regarding to HLA class I, the combining A, B, and C allele results were generated from POLYSOLVER, OpiType, and xHLA. HLA‐HD, SOAP‐HLA, and xHLA were utilized for creating ensemble results on DRB1, DQB1, DPB1, and DQA1. As shown in Fig. 4A,B, the accuracy of ensemble results on HLA class I genes (A, B, C) and HLA class II genes (DRB1, DQB1, DPB1 and DQA1) would reach 0.981 and 0.907, respectively. Taking together, the ensemble results of the top 3 tools are superior to individual ones. It is highly recommended to generate reliable HLA results based on the concordance alleles integrated from multiple tools.

### 3.6 The impact of HLA on survival

**Fig. 5:**
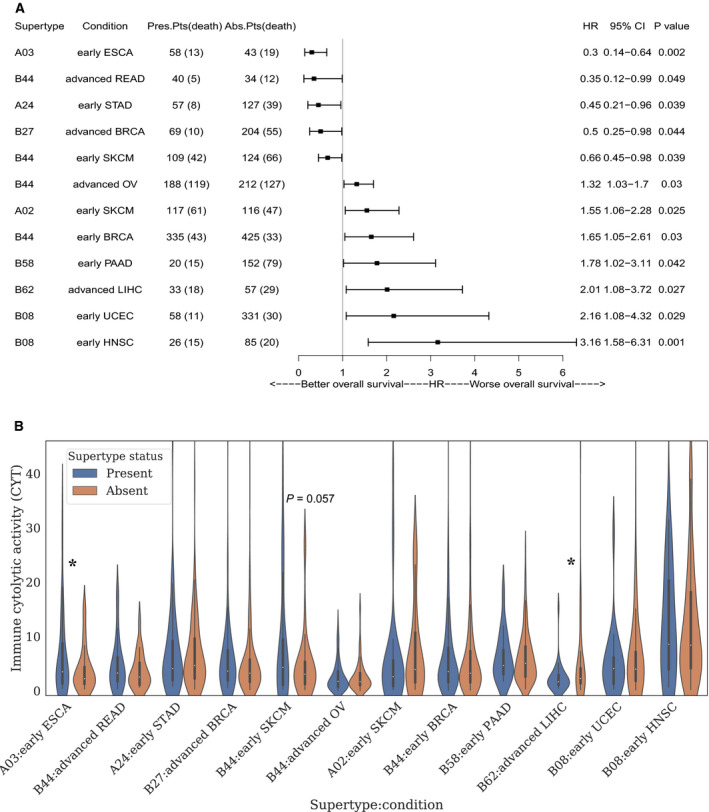
The survival impact of HLA alleles in TCGA cancer conditions. (A) Forest plot of univariate CoxPH analyses on OS. According to the supertypes status, either ‘present’ or ‘absent’ were assigned to each patient. Only the supertypes with significant influence on OS were displayed. (B) Immune cytolytic activity analysis on HLA supertypes under specific cancer conditions mentioned in Fig. 4A. The patients with specific HLA supertype were marked in blue. While, orange bar represents the supertype‐absent patients. Violin plot and one‐tailed t test were generated by python package seaborn and statsmodels, respectively. “*” indicate significant with t test *P* value ≤ 0.05.

The univariate CoxPH regression model was firstly utilized to analyze the impact of specific HLA supertypes on OS in TCGA cohort. In this study, HLA supertypes are defined as the groups of HLA alleles sharing specific amino residues at anchor positions of the B and F pockets in the peptide‐binding region. The HLA alleles within same supertypes were expected to have similar presentation ability. In detail, regarding to each specific HLA supertype, two subgroups could be defined based on allele present or absent on the patients. Subsequently, risk comparison was implemented among the two subgroups. As shown in Fig. 5A, eight HLA supertypes were observed with a significant impact on survival (Wald test *P* < 0.05, see full list in Table S6). Each record with hazard ratio (HR) < 1 means the specific HLA supertype could serve as a beneficial factor under that condition. However, records with HR > 1 probably indicate that such specific HLA supertype increase the death risk on the cancer condition. It seems that the impact of HLA on OS may be disease‐specific. The same HLA supertype may generate distinct influence among cancer conditions. For example, different survival impact of B44 was found in early‐stage skin cutaneous melanoma (SKCM), advanced stage rectum adenocarcinoma (READ), advanced stage ovarian serous cystadenocarcinoma (OV), and early‐stage breast invasive carcinoma (BRCA). Specifically, better survival was observed in the B44‐present rather than B44‐absent on the previous two cancer conditions (early‐stage SKCM: HR = 0.66, Wald test *P* = 0.039; advanced stage READ: HR = 0.35, Wald test *P* = 0.049). On the contrary, B44‐present patients showed worse survival in advanced stage OV and early‐stage BRCA (advanced stage OV: HR = 1.32, Wald test *P* = 0.03; early‐stage BRCA: HR = 1.66, Wald test *P* = 0.03).

Since many cofounding factors may interact with HLA allele on OS, a more precise multivariate CoxPH model analysis was further performed on the HLA supertype and tumors as presented in Fig. 5A. The age at initial diagnosis of tumor may played a dominant harmful role in advanced stage OV and early‐stage BRCA (advanced stage OV: HR = 1.59, Wald test *P* = 0.001; early‐stage BRCA: HR = 3.50, Wald test *P* = 7.18E‐06), comparing with HLA supertype B44 (advanced stage OV: HR = 1.26, Wald test *P* = 0.112; early‐stage BRCA: HR = 1.55, Wald test *P* = 0.104), while the beneficial effect of B44 on survival was still observed in early‐stage SKCM and advanced stage READ. Specially, no significant survival impact of TMB or age was detected in advanced stage READ (Table 1). It is interesting that the beneficial effect of B44 on SKCM survival is exactly consistent with B44 on ICBs treated melanoma survival [9]. Since ICBs is not the dominant treatment in TCGA SKCM cohort, it seems that the beneficial effect of B44 on the SKCM survival is independent of ICBs treatment. In addition, B62 was reported to show negative correlation with survival of ICBs treated melanoma in Chowell's study [9]. Such harmful effect of B62 on survival was also observed in TCGA advanced liver hepatocellular carcinoma (LIHC) cohort.

**Table 1:** The multivariate CoxPH analysis of HLA supertypes and tumors in Fig. 5A.

Multivariate analysis		Num. Pts in condition 1 (death)	Num. Pts in condition 2 (death)	HR	CI_95%	*P* value
Early‐stage ESCA	Condition 1 vs condition 2					
A03	Present vs absent	47 (11)	33 (14)	0.27	0.11–0.67	0.005
TMB	High vs low	41 (15)	39 (10)	1.89	0.82–4.34	0.135
Age	≥ 60 vs < 60	39 (13)	41 (12)	0.79	0.34–1.83	0.577
Advanced stage READ	Condition 1 vs condition 2					
B44	Present vs absent	23 (2)	25 (10)	0.15	0.03–0.71	0.017
TMB	High vs low	26 (8)	22 (4)	0.84	0.23–3.08	0.795
Age	≥ 60 vs < 60	30 (11)	18 (1)	4	0.50–31.73	0.19
Early‐stage stomach adenocarcinoma	Condition 1 vs condition 2					
A24	Present vs absent	40 (5)	102 (32)	0.39	0.15–1.01	0.052
TMB	High vs low	67 (16)	75 (21)	0.78	0.39–1.54	0.469
Age	≥ 60 vs < 60	103 (29)	39 (8)	1.54	0.68–3.50	0.299
Advanced stage BRCA	Condition 1 vs condition 2					
B27	Present vs absent	53 (6)	143 (36)	0.3	0.13–0.73	0.008
TMB	High vs low	102 (27)	94 (15)	2.58	1.35–4.93	0.004
Age	≥ 60 vs < 60	87 (21)	109 (21)	1.65	0.89–3.06	0.114
Early‐stage SKCM	Condition 1 vs condition 2					
B44	Present vs absent	90 (34)	97 (53)	0.58	0.37–0.90	0.015
A02	Present vs absent	94 (51)	93 (36)	1.67	1.09–2.58	0.02
TMB	High vs low	98 (42)	89 (45)	0.62	0.40–0.97	0.035
Age	≥ 60 vs < 60	98 (45)	89 (42)	2.53	1.55–4.12	1.99E‐04
Advanced stage OV	Condition 1 vs condition 2					
B44	Present vs absent	142 (91)	172 (103)	1.26	0.95–1.67	0.112
TMB	High vs low	161 (91)	153 (103)	0.74	0.55–0.98	0.034
Age	≥ 60 vs < 60	145 (103)	169 (91)	1.59	1.20–2.11	0.001
Early‐stage BRCA	Condition 1 vs condition 2					
B44	Present vs absent	257 (32)	321 (26)	1.55	0.91–2.62	0.104
TMB	High vs low	298 (26)	280 (32)	0.67	0.40–1.14	0.142
Age	≥ 60 vs < 60	263 (38)	315 (20)	3.5	2.03–6.06	7.19E‐06
Early‐stage PAAD	Condition 1 vs condition 2					
B58	Present vs absent	18 (13)	123 (61)	1.63	0.89–3.00	0.113
TMB	High vs low	79 (48)	62 (26)	1.56	0.96–2.53	0.072
Age	≥ 60 vs < 60	100 (57)	41 (17)	1.52	0.88–2.65	0.135
Advanced stage LIHC	Condition 1 vs condition 2					
B62	Present vs absent	28 (16)	44 (24)	1.95	0.94–4.01	0.071
TMB	High vs low	40 (22)	32 (18)	0.92	0.47–1.81	0.815
Age	≥ 60 vs < 60	37 (20)	35 (20)	0.92	0.47–1.82	0.817
Early‐stage HNSC	Condition 1 vs condition 2					
A24	Present vs absent	18 (10)	59 (14)	2.42	0.99–5.92	0.053
TMB	High vs low	39 (15)	38 (9)	1.23	0.52–2.87	0.639
Age	≥ 60 vs < 60	46 (18)	31 (6)	1.88	0.72–4.91	0.197
Early‐stage UCEC	Condition 1 vs condition 2					
B08	Present vs absent	42 (7)	247 (25)	2.03	0.86–4.78	0.105
TMB	High vs low	147 (10)	142 (22)	0.47	0.22–0.99	0.046
Age	≥ 60 vs < 60	190 (27)	99 (5)	2.76	1.06–7.19	0.038

The antigen presentation role of HLA might influence survival through increasing immune response. Based on such assumption, we further investigate the corresponding CYT among the HLA supertypes‐present/absent cohorts in different cancer conditions as displayed in Fig. 5A. The average CYT of B44‐present patients in early‐stage SKCM, advanced stage READ were slightly higher than that of B44‐absent patients (Fig. 5B, Table S7). Specifically, such difference trend reached *P* value at 0.057 in early‐stage SCKM, which is very close to the statistic confidence cutoff. However, no difference of mean CYT was detected among the patients in early‐stage BRCA and advanced stage OV regardless of B44 status. In addition, since the age was detected as a dominant risk factor in the multivariate CoxPH regression, such observed harmful impact of B44 on the two cancer conditions was probably not caused by immune response. It should be noted that nearly 88.22% OV patients in TCGA received carboplatin treatment. Since chemotherapy is most likely to weaken the immune system, such survival risk observed in TCGA OV cohorts is probably not caused by HLA alleles. We further checked the survival impact of CYT on the four cancer conditions in TCGA cohorts (see full list in Fig. S1). High‐CYT patients displayed extended survival in early‐stage SKCM with HR = 0.33 (CI: 0.1–1.1, *P* = 0.067). No distinct impact of CYT on survival was found in other three cancer conditions. (advanced stage READ: HR = 1.75, CI = 0.49–6.27, *P* = 0.393; early‐stage BRCA: HR = 0.80, CI = 0.51–1.27, *P* = 0.349; advanced stage OV: HR = 0.95, CI = 0.71–1.26, *P* = 0.705). It may indicate that immune signaling does not serve a dominant role on survival in such three cancer conditions. Regarding to B62, the CYT of B62‐present patients in advanced stage LIHC is significantly lower than that of B62‐absent patients (Fold change = 0.49, *P* value = 0.033).

Since HLA allele‐specific LOH event also indicates a type of allele‐absent conditions, it could precisely describe the allele status in the patient. We further evaluated the survival impact between the B44‐present patients without any B44‐LOH events and patients with either B44‐absent or B44‐LOH using both univariate and multivariate CoxPH analyses, the HR value was achieved at 0.64 (Wald test *P* = 0.025) and 0.63 (Wald test *P* = 0.025) in early‐stage SKCM, respectively (Fig. S2A, Table S8). In addition, the mean CYT of B44‐present group is significantly greater than B44‐LOH/B44‐absent group (Fold change = 1.83, *P* = 0.033) (Fig. S2B, Table S9). It indicates that CYT may partially clarify the survival impact of HLA alleles.

### 3.7 The ‘Gun‐Bullet’ model for interpreting the impact of HLA on survival

In canonical immune process, the immune signaling is stimulated by the recognition of TMB‐derived antigen and HLA allele [32, 33]. TMB and HLA allele were both essential contributing for immune signaling that finally lead to CYT of effector T cells. It is quite controversial on the impact of the HLA in survival, since opposite conclusions were generated in two previous studies. In Chowell's study [9], HLA supertype B44 was associated with extended survival in patients with melanoma receiving ICBs from two independent cohorts. However, no HLA supertypes were observed with significant impact on survival, regarding to an ICB‐treated NSCLC cohort in M. D. Anderson Cancer Center [10]. How can we interpret such disease‐specific impact of HLA supertype on survival? The distinct mutational signatures of amino acids across cancer types may provide clues on such issue [34]. In the molecular level, HLA‐B44 share a preference for glutamic acid (E) at anchor position 2, which is exactly match the most enriched amino acid mutations (G>E) in melanoma. It may indicate that HLA‐B44 prefers to presenting such ‘E’ enriched mutant peptides in melanoma. The subsequently stimulated immune signaling is the ultimate effector that response for the survival extension of patients receiving ICBs. CYT, deriving from the expression of GZMA and PRF1 that specifically released by effector T cells, could well mimic the intratumoral cytolytic T‐cell activity in microenvironment. According to Rooney's study [11], high CYT was proofed as a beneficial factor for modest but significant pan‐cancer survival in previous TCGA pan‐cancer study. Moreover, in the two independent ICB‐treated melanoma cohorts [23, 35], the significant extended OS were observed in the high‐CYT group (median OS in Van Allen's cohort 27 vs 7 m, *P* = 0.0011; median OS in Tuba's cohort 732 vs 506 d, *P* = 0.0006, see details in Fig. S3). Furthermore, we also found that significant higher CYT was detected in DCB group than NDB group from two independent NSCLC cohorts under anti‐PD1 therapy. Especially, in Jae‐Won Cho's cohort [28], the median OS in high‐CYT group is significant longer than low‐CYT group (median OS: 14.1 vs 3.1 m, *P* = 0.0048) (Fig. S4). Therefore, CYT may provide us novel insights on the survival impact of HLA alleles.

**Fig. 6:**
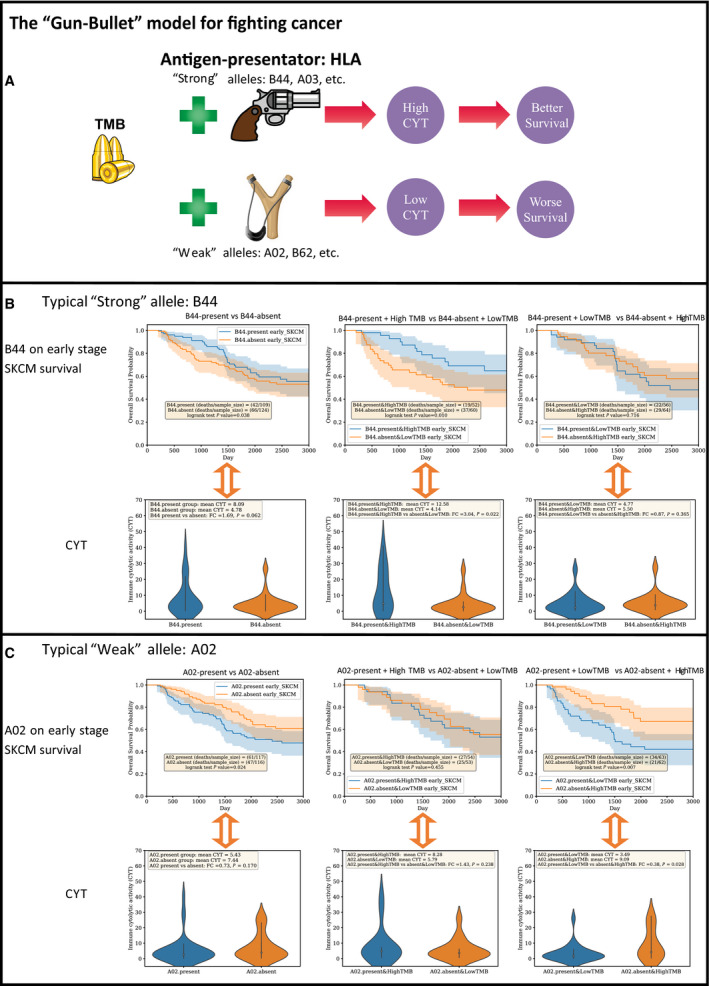
The “Gun‐Bullet” model for illustrating the survival impact of HLA. (A). The brief chart illustrates how TMB and HLA exert the impact of survival through CYT. The impact of HLA alleles on OS may partially due to CYT. A strong HLA plus a high TMB could stimulate intensive immune response (CYT), leading to a better survival. Stronger HLA alleles are more able to present antigens (TMB) and subsequently induce higher CYT. The relationship between TMB and HLA allele is quite similar to the bullet and gun. A powerful gun plus high quantity bullets could maximize the force. The clipart in the figure were downloaded from http://clipart‐library.com with the licence of copyright free for non‐commercial use. (B). The typical ‘strong’ HLA B44 in early stage SKCM survival. Kaplan‐Meier survival analysis and corresponding CYT comparison were implemented for the following three cohort pairs: B44‐present vs B44‐absent; B44‐present&HighTMB vs B44‐absent&LowTMB; B44‐present&LowTMB vs B44‐absent&HighTMB. (C). The typical ‘weak’ HLA A02 in early stage SKCM survival. Survival analysis and corresponding CYT comparison were implemented for the following three cohort pairs: A02‐present vs A02‐absent; A02‐present&HighTMB vs A02‐absent&LowTMB; A02‐present&LowTMB vs A02‐absent&HighTMB. Survival curves and violin plot were generated by python package lifelines and seaborn, respectively. One‐tailed t test was implemented by R. High and Low TMB groups were determined by whether the TMB values higher or lower than the median TMB of the specific cancer condition. For example, the sample size of patients in early stage SKCM TCGA cohort is 237. The median TMB in early stage SKCM is 9.521. (see the full median TMB information in Table S10).

Herein, we proposed a ‘Gun‐Bullet’ model for interpreting the impact of HLA on survival. As shown in Fig. 6A, the impact of HLA and TMB on CYT is quite similar to the relationship between gun and bullets on force. A powerful gun plus high quantity bullets could maximize the force. Similarly, a strong HLA allele plus a high TMB could stimulate intensive immune response (CYT), leading to longer survival. ‘Stronger’ HLA alleles are more able to present antigens (TMB) and subsequently induce higher CYT. As shown in Fig. 6B, regarding to a typical ‘strong’ HLA supertype B44 in early‐stage SKCM, the maximum distinct CYT and OS were found between B44‐present and high‐TMB group and B44‐absent & Low‐TMB group, while no difference on both CYT and OS was observed in B44‐present and low‐TMB group and B44‐absent and high‐TMB group. Since much less antigens are available in low‐TMB group, it is unexpected to achieve high CYT in such B44‐present patients. On the contrary, the ‘weak’ allele A02‐present and low‐TMB patients displayed the minimum CYT and short OS. No obvious difference on CYT and OS was found between A02‐present and high‐TMB group and A02‐absent and low‐TMB group (Fig. 6C). Although much more antigens were available for A02‐presenting in high‐TMB group, no dramatically increase of CYT could occur due to the weak antigen presentation ability of A02. That means TMB is a critical factor for the immune response that regulated by HLA allele. The patients with a ‘strong’ HLA allele as well as a high TMB were likely to induce a strong CYT and a better survival. However, since insufficient antigens could be generated for HLA presentation in cancers with low‐TMB background, HLA may be not qualified as an independent prognostic biomarker for survival. At least, the signal of HLA impact on survival is much weaker in cancers with a low‐TMB background than that of a high‐TMB background. Melanoma had always been reported as a typical high‐TMB cancer with median TMB larger than 14 in several large pan‐cancer cohorts, including Foundation cohort [36], TCGA cohort [37], MSK‐IMPACT cohort [38], and Yarchoan's cohort [39]. NSCLC displayed much lower TMB in the studies mentioned above. This may partially explain the debates of HLA as a prognostic biomarker occurred in Chowell's [9] melanoma cohort and Negrao's [10] NSCLC cohort.

The ‘Gun‐Bullet’ model can be used to clarify the survival impact of HLA in ICB‐treated cohort as well. As we mentioned above, a higher CYT generally indicates a longer survival in two melanoma cohorts [23, 35] and two NSCLC cohorts [28, 29] under ICB treatment. In addition, B44 and TMB were detected with positive correlation with survival in Chowell's cohort [9]. Furthermore, the effect of B44 on extended survival was greater when TMB was also considered. (*P* = 0.00003, median OS: 23.7 vs 8.2 m) (Fig. S5). Since the ensemble effect of B44 and TMB on survival was greater than any individual one, it may indicate that complementary roles of B44 and TMB on survival.

However, B44‐present patients did not display any survival advantage, comparing with the B44‐absent patients in Van Allen's cohort [23] (Fig. S6A). The same situation was also detected for TMB as an individual prognostic biomarker (Fig. S6B), although the trend of clinical benefit was observed in survival curve between B44‐present and high‐TMB group and B44‐absent and low‐TMB group (Fig. S6C). However, it did not achieve statistical significance. Regarding to the ‘Gun‐Bullet’ model, such situation may be caused by the similar CYT among these comparison conditions. As expected, no significant higher CYT was found in B44‐present group (Fig. S6D), high‐TMB group (Fig. S6E), or B44‐present and high‐TMB group (Fig. S6F). Since CYT may serve as an underlying mechanism in ‘Gun‐Bullet’ model for interpreting the survival impact of HLA and TMB, such phenomena are explainable. No survival advantage could be observed among the patients with statistically equivalent CYT, regardless of the TMB or B44 status.

## 4 Discussion

In this study, we have evaluated eight frequently used and publicly available NGS‐based HLA genotyping tools on TCGA WES data. Since no golden standard HLA dataset is available, constructing a HLA benchmark dataset is the critical point ahead of performance evaluation. With the assumption that the ‘True’ HLA alleles are likely to be frequently reported by different HLA genotyping tools, a TCGA HLA benchmark dataset covering seven major HLA genes: HLA‐A, B, C, DRB1, DPB1, DQA1, and DQB1, was generated based on the concordance allele processing. Actually, we tried to implement a comprehensive performance evaluation on WES‐based HLA genotyping tools as more as possible. More than 11 tools were included in the original study design, and some of them had been discarded in pilot test due to different issues. For example, HLAscan [40] is not supported for NGS data with reads length < 76 bp. That means more than 41% TCGA WES data (reads length < 76 bp) were unable to generate the HLA genotyping results. However, the source code of PHLAT [41] is not convenient to access. HLA‐PRG‐LA [42] utilizes the precise graph alignment during HLA genotyping which requires extremely high computational resource. In our pilot test, it costs nearly 4 h to process a WES data (see the running time in Table S11). That makes it unsuitable for performance assessment on the whole TCGA dataset. Since such limitations exist on tools selection, the tested eight tools may not represent the highest accuracy achieved by existing NGS‐based HLA genotyping tools. However, they can be treated as the robust and efficient methods to generate the HLA genotyping results.

Distinct error patterns were detected in each HLA genotyping tools. Such error may not derive from the allele inference models utilized in the tools. The outdated HLA allele database used in the initiate reads alignment would be one of the issues. For example, SOAP‐HLA was failed on calling DRB1:08, DRB1:11 alleles at the 4‐digit resolution, since the background allele database in SOAP‐HLA was updated 5 years ago. Although the background allele database is important for accurate genotyping, the documents on updating the database are always lacking in most of the tools. It would be a typical issue in current NGS‐based HLA genotyping tools.

CYT was proposed as the underlying mechanism to interpret the survival impact of HLA alleles in the ‘Gun‐Bullet’ model for fighting cancer. Since CD8+ T cell and NK cell were preferred immune cells for killing cancer in the microenvironment, it is important to investigate whether CYT detected in bulk tumor tissue could reflect the cell fractions of CD8+ T cell and NK cell in TILs. Referring to the deconvolution‐based approach in CIBERSORT [43] with absolute mode, we retrieved the cell fraction of 22 immune cell types of each TCGA sample (downloaded at http://timer.cistrome.org/). As shown in Fig. S7, CYT was highly correlated with the cell fractions of CD8+ T cell and activated NK cell evaluated in the corresponding bulk tumor tissues (Pearson correlation coefficient = 0.73, *P* < 1E‐20), while extremely low correlation was detected between the cell fraction of resting NK cell and CYT (Pearson correlation coefficient = 0.07). It indicates that CYT detected in the bulk tumor tissue could probably severed as an indicator of CD8+ T cell and activated NK cell in TILs. It is biologically reasonable to use CYT to interpret the survival impact of HLA alleles.

The clinical benefit effect of CYT was verified in 4 independent NSCLC and melanoma cohorts under ICB treatment as well as TCGA pan‐cancer cohorts. In addition, the relationship among B44, TMB, and CYT on survival has been proved in TCGA early‐stage SKCM cohort. It still lacks of a positive case from ICB‐treated cohorts. In Allen's [23] melanoma cohort under anti‐CTLA4 therapy, no significant higher CYT was detected in B44‐present group, high‐TMB group, and B44‐present and High‐TMB group. Therefore, no significant extended survival could be observed in any of these groups. It could be treated as a negative case that explained by ‘Gun‐Bullet’ model. In Chowell's cohort [9], the extended survival was greater in B44‐present and high‐TMB group than each isolated situation. However, the CYT information is not available in such cohort. More ICB‐treated cohorts with both RNA‐seq (for CYT calculation) and WES (for HLA genotyping and TMB) data were needed to verified the ‘Gun‐Bullet’ model.

## 5 Conclusions

In summary, we established an up to now most reliable TCGA HLA benchmark dataset, composing of concordance alleles generated from eight prevalently used HLA genotyping tools. Each HLA genotyping tool displayed specific error pattern, and it is better to generate reliable results with multiple tools. Regarding to the survival analysis, HLA alleles show distinct survival impact among cancers. CYT, as the readout of immune signaling, could partially bridge the HLA allele and survival. According to the ‘Gun‐Bullet’ model, a strong HLA allele plus a high TMB could stimulate intensive immune CYT, leading to an extended survival. Although TMB is a critical factor in the stimulating immune response, HLA allele could amplify or attenuate the effect of TMB. In most of cancers with the low‐TMB baseline, HLA allele is unlikely to serve as a prognostic biomarker on survival, since minimum antigens were provided for HLA presenting. Similar to the relationship between gun and bullet, the integrative effect of TMB and HLA allele was likely to stimulate immune response and influence survival. It indicated that incorporating TMB with HLA genotype helps to improve the survival prediction compared to HLA genotyping alone.

## Conflict of interest

The authors declare no conflict of interest.

## Author contributions

QL and HY conceived the study. CZ and KC obtained the WES data of TCGA cohort and RNA‐seq data of ICB cohorts. CZ, XYL, and HY processed the samples and performed the whole analysis. BH provided useful discussions on benchmarking HLA genotyping and the ‘Gun‐Bullet’ model. CZ, HY, and QL wrote the manuscript with assistance from other authors.

## Supporting information


**Fig. S1.** The survival impact of CYT in TCGA cancer conditions.

**Fig. S2.** The survival impact of HLA alleles (with consideration of LOH status) in TCGA cancer conditions.

**Fig. S3.** The survival impact of CYT in two ICB‐treated melanoma cohorts.

**Fig. S4.** The survival impact of CYT in two ICB‐treated NSCLC cohorts.

**Fig. S5.** The survival curve in Chowell's ICB‐treated melanoma cohort.

**Fig. S6.** The survival plot and corresponding violin plot on CYT in Van Allen's melanoma cohort.

**Fig. S7**. The correlation between CYT and cell fraction evaluated by CIBERSORT in absolute model.

**Table S1.** The clinical information of cohorts under ICB therapy.

**Table S2.** The statistics of HLA alleles in TCGA benchmark dataset.

**Table S3.** Error rate of HLA Class I/II alleles.

**Table S4.** The HLA class I error calling information in the eight tools.

**Table S5.** The HLA class II error calling information in the six tools.

**Table S6.** The HR in all of the TCGA cancer conditions generated by univariate CoxPH regression.

**Table S7.** CYT of the HLA supertypes‐present/absent cohorts in different cancer conditions.

**Table S8.** The multivariable CoxPH analysis of HLA alleles and tumors in Fig. S2A.

**Table S9.** CYT of the HLA supertypes‐LOH/NotLOH cohorts in different cancer conditions.

**Table S10.** The median TMB and CYT in each cancer condition.

**Table S11.** The running time of tested HLA genotyping tools.

## Data Availability

Whole exome sequencing data were retrieved from TCGA database through dbGap with accession number phs000178.v11.p8. Raw RNA‐seq data for the Tuba *et al*. cohort are obtained from the European Nucleotide Archive (ENA), under accession number PRJEB23709. And RNA‐seq data for the Van Allen *et al*. cohort are available on the Sequence Read Archive under accession number phs000452.v2.p1. RNA‐seq data for Jae‐Won Cho's NSCLC cohort and Jeong Yeon Kim's NSCLC cohort were downloaded from Gene Expression Omnibus with access number GSE126044 and GSE135222, respectively. Code for analyses and HLA genotyping results are available at https://github.com/biolxy/TCGA_HLA_benchmark.

## References

[1] Robinson J , Halliwell JA , Hayhurst JD , Flicek P , Parham P & Marsh SG (2015) The IPD and IMGT/HLA database: allele variant databases. Nucleic Acids Res 43, D423–D431. 25414341 10.1093/nar/gku1161 PMC4383959

[2] Bauer DC , Zadoorian A , Wilson LOW , Melbourne Genomics Health Alliance & Thorne NP (2018) Evaluation of computational programs to predict HLA genotypes from genomic sequencing data. Brief Bioinform 19, 179–187. 27802932 10.1093/bib/bbw097 PMC6019030

[3] Shukla SA , Rooney MS , Rajasagi M , Tiao G , Dixon PM , Lawrence MS , Stevens J , Lane WJ , Dellagatta JL , Steelman S *et al*. (2015) Comprehensive analysis of cancer‐associated somatic mutations in class I HLA genes. Nat Biotechnol 33, 1152–1158. 26372948 10.1038/nbt.3344 PMC4747795

[4] Szolek A , Schubert B , Mohr C , Sturm M , Feldhahn M & Kohlbacher O (2014) OptiType: precision HLA typing from next‐generation sequencing data. Bioinformatics 30, 3310–3316. 25143287 10.1093/bioinformatics/btu548 PMC4441069

[5] Lee H & Kingsford C (2018) Accurate assembly and typing of HLA using a Graph‐Guided Assembler Kourami. Methods Mol Biol 1802, 235–247. 29858814 10.1007/978-1-4939-8546-3_17

[6] Marty Pyke R , Thompson WK , Salem RM , Font‐Burgada J , Zanetti M & Carter H (2018) Evolutionary pressure against MHC Class II binding cancer mutations. Cell 175, 416–428.e413. 30245014 10.1016/j.cell.2018.08.048 PMC6482006

[7] Marty R , Kaabinejadian S , Rossell D , Slifker MJ , van de Haar J , Engin HB , de Prisco N , Ideker T , Hildebrand WH , Font‐Burgada J *et al*. (2017) MHC‐I genotype restricts the oncogenic mutational landscape. Cell 171, 1272–1283.e1215. 29107334 10.1016/j.cell.2017.09.050 PMC5711564

[8] McGranahan N , Rosenthal R , Hiley CT , Rowan AJ , Watkins TBK , Wilson GA , Birkbak NJ , Veeriah S , Van Loo P , Herrero J *et al*. (2017) Allele‐specific HLA loss and immune escape in lung cancer evolution. Cell 171, 1259–1271.e1211. 29107330 10.1016/j.cell.2017.10.001 PMC5720478

[9] Chowell D , Morris LGT , Grigg CM , Weber JK , Samstein RM , Makarov V , Kuo F , Kendall SM , Requena D , Riaz N *et al*. (2018) Patient HLA class I genotype influences cancer response to checkpoint blockade immunotherapy. Science 359, 582–587. 29217585 10.1126/science.aao4572 PMC6057471

[10] Negrao MV , Lam VK , Reuben A , Rubin ML , Landry LL , Roarty EB , Rinsurongkawong W , Lewis J , Roth JA , Swisher SG *et al*. (2019) PD‐L1 expression, tumor mutational burden and cancer gene mutations are stronger predictors of benefit from immune checkpoint blockade than HLA class I genotype in non‐small cell lung cancer. J Thorac Oncol 14, 1021–1031. 30780001 10.1016/j.jtho.2019.02.008

[11] Rooney MS , Shukla SA , Wu CJ , Getz G & Hacohen N (2015) Molecular and genetic properties of tumors associated with local immune cytolytic activity. Cell 160, 48–61. 25594174 10.1016/j.cell.2014.12.033 PMC4856474

[12] Kawaguchi S , Higasa K , Shimizu M , Yamada R & Matsuda F (2017) HLA‐HD: an accurate HLA typing algorithm for next‐generation sequencing data. Human Mutat 38, 788–797. 10.1002/humu.23230 28419628

[13] Xie C , Yeo ZX , Wong M , Piper J , Long T , Kirkness EF , Biggs WH , Bloom K , Spellman S , Vierra‐Green C *et al*. (2017) Fast and accurate HLA typing from short‐read next‐generation sequence data with xHLA. Proc Natl Acad Sci USA 114, 8059–8064. 28674023 10.1073/pnas.1707945114 PMC5544337

[14] Nariai N , Kojima K , Saito S , Mimori T , Sato Y , Kawai Y , Yamaguchi‐Kabata Y , Yasuda J & Nagasaki M (2015) HLA‐VBSeq: accurate HLA typing at full resolution from whole‐genome sequencing data. BMC Genom 16 (Suppl 2), S7. 10.1186/1471-2164-16-S2-S7 PMC4331721 25708870

[15] Lee H & Kingsford C (2018) Kourami: graph‐guided assembly for novel human leukocyte antigen allele discovery. Genome Biol 19, 16. 29415772 10.1186/s13059-018-1388-2 PMC5804087

[16] Sidney J , Peters B , Frahm N , Brander C & Sette A (2008) HLA class I supertypes: a revised and updated classification. BMC Immunol 9, 1. 18211710 10.1186/1471-2172-9-1 PMC2245908

[17] Ambrose AR , Hazime KS , Worboys JD , Niembro‐Vivanco O & Davis DM (2020) Synaptic secretion from human natural killer cells is diverse and includes supramolecular attack particles. Proc Natl Acad Sci USA 117, 23717–23720. 32900953 10.1073/pnas.2010274117 PMC7519227

[18] Cao X , Cai SF , Fehniger TA , Song J , Collins LI , Piwnica‐Worms DR & Ley TJ (2007) Granzyme B and perforin are important for regulatory T cell‐mediated suppression of tumor clearance. Immunity 27, 635–646. 17919943 10.1016/j.immuni.2007.08.014

[19] Narayanan S , Kawaguchi T , Yan L , Peng X , Qi Q & Takabe K (2018) Cytolytic activity score to assess anticancer immunity in colorectal cancer. Ann Surg Oncol 25, 2323–2331. 29770915 10.1245/s10434-018-6506-6 PMC6237091

[20] Roufas C , Chasiotis D , Makris A , Efstathiades C , Dimopoulos C & Zaravinos A (2018) The expression and prognostic impact of immune cytolytic activity‐related markers in human malignancies: a comprehensive meta‐analysis. Front Oncol 8, 27. 29515971 10.3389/fonc.2018.00027 PMC5826382

[21] Devarakonda S , Rotolo F , Tsao MS , Lanc I , Brambilla E , Masood A , Olaussen KA , Fulton R , Sakashita S , McLeer‐Florin A *et al*. (2018) Tumor mutation burden as a biomarker in resected non‐small‐cell lung cancer. J Clin Oncol 36, 2995–3006. 30106638 10.1200/JCO.2018.78.1963 PMC6804865

[22] Melendez B , Van Campenhout C , Rorive S , Remmelink M , Salmon I & D'Haene N (2018) Methods of measurement for tumor mutational burden in tumor tissue. Transl Lung Cancer Res 7, 661–667. 30505710 10.21037/tlcr.2018.08.02 PMC6249625

[23] Van Allen EM , Miao D , Schilling B , Shukla SA , Blank C , Zimmer L , Sucker A , Hillen U , Foppen MHG , Goldinger SM *et al*. (2015) Genomic correlates of response to CTLA‐4 blockade in metastatic melanoma. Science 350, 207–211. 26359337 10.1126/science.aad0095 PMC5054517

[24] Gide TN , Quek C , Menzies AM , Tasker AT , Shang P , Holst J , Madore J , Lim SY , Velickovic R & Wongchenko M (2019) Distinct immune cell populations define response to anti‐PD‐1 monotherapy and anti‐PD‐1/anti‐CTLA‐4 combined therapy. Cancer Cell 35, 238–255.e236. 30753825 10.1016/j.ccell.2019.01.003

[25] Wei Z , Zhou C , Zhang Z , Guan M , Zhang C , Liu Z & Liu Q (2019) The landscape of tumor fusion neoantigens: a pan‐cancer analysis. iScience 21, 249–260. 31677477 10.1016/j.isci.2019.10.028 PMC6838548

[26] Zhang Z , Zhou C , Tang L , Gong Y , Wei Z , Zhang G , Wang F , Liu Q & Yu J (2020) ASNEO: identification of personalized alternative splicing based neoantigens with RNA‐seq. Aging 12, 14633–14648. 32697765 10.18632/aging.103516 PMC7425491

[27] Zhou C , Wei Z , Zhang Z , Zhang B , Zhu C , Chen K , Chuai G , Qu S , Xie L , Gao Y *et al*. (2019) pTuneos: prioritizing tumor neoantigens from next‐generation sequencing data. Genome Med 11, 67. 31666118 10.1186/s13073-019-0679-x PMC6822339

[28] Cho JW , Hong MH , Ha SJ , Kim YJ , Cho BC , Lee I & Kim HR (2020) Genome‐wide identification of differentially methylated promoters and enhancers associated with response to anti‐PD‐1 therapy in non‐small cell lung cancer. Exp Mol Med 52, 1550–1563. 32879421 10.1038/s12276-020-00493-8 PMC8080767

[29] Kim JY , Choi JK & Jung H (2020) Genome‐wide methylation patterns predict clinical benefit of immunotherapy in lung cancer. Clin Epigenetics 12, 119. 32762727 10.1186/s13148-020-00907-4 PMC7410160

[30] Dobin A , Davis CA , Schlesinger F , Drenkow J , Zaleski C , Jha S , Batut P , Chaisson M & Gingeras TR (2013) STAR: ultrafast universal RNA‐seq aligner. Bioinformatics 29, 15–21. 23104886 10.1093/bioinformatics/bts635 PMC3530905

[31] Li B & Dewey CN (2011) RSEM: accurate transcript quantification from RNA‐Seq data with or without a reference genome. BMC Bioinformatics 12, 323. 21816040 10.1186/1471-2105-12-323 PMC3163565

[32] Guermonprez P , Valladeau J , Zitvogel L , Thery C & Amigorena S (2002) Antigen presentation and T cell stimulation by dendritic cells. Annu Rev Immunol 20, 621–667. 11861614 10.1146/annurev.immunol.20.100301.064828

[33] Matsushita H , Sato Y , Karasaki T , Nakagawa T , Kume H , Ogawa S , Homma Y & Kakimi K (2016) Neoantigen load, antigen presentation machinery, and immune signatures determine prognosis in clear cell renal cell carcinoma. Cancer Immunol Res 4, 463–471. 26980598 10.1158/2326-6066.CIR-15-0225

[34] Havel JJ , Chowell D & Chan TA (2019) The evolving landscape of biomarkers for checkpoint inhibitor immunotherapy. Nat Rev Cancer 19, 133–150. 30755690 10.1038/s41568-019-0116-x PMC6705396

[35] Gide TN , Quek C , Menzies AM , Tasker AT , Shang P , Holst J , Madore J , Lim SY , Velickovic R , Wongchenko M *et al*. (2019) Distinct immune cell populations define response to anti‐PD‐1 monotherapy and anti‐PD‐1/Anti‐CTLA‐4 combined therapy. Cancer Cell 35, 238–255.e236. 30753825 10.1016/j.ccell.2019.01.003

[36] Chalmers ZR , Connelly CF , Fabrizio D , Gay L , Ali SM , Ennis R , Schrock A , Campbell B , Shlien A , Chmielecki J *et al*. (2017) Analysis of 100,000 human cancer genomes reveals the landscape of tumor mutational burden. Genome Med 9, 34. 28420421 10.1186/s13073-017-0424-2 PMC5395719

[37] Alexandrov LB , Nik‐Zainal S , Wedge DC , Aparicio SA , Behjati S , Biankin AV , Bignell GR , Bolli N , Borg A , Borresen‐Dale AL *et al*. (2013) Signatures of mutational processes in human cancer. Nature 500, 415–421. 23945592 10.1038/nature12477 PMC3776390

[38] Zehir A , Benayed R , Shah RH , Syed A , Middha S , Kim HR , Srinivasan P , Gao J , Chakravarty D , Devlin SM *et al*. (2017) Mutational landscape of metastatic cancer revealed from prospective clinical sequencing of 10,000 patients. Nat Med 23, 703–713. 28481359 10.1038/nm.4333 PMC5461196

[39] Yarchoan M , Hopkins A & Jaffee EM (2017) Tumor mutational burden and response rate to PD‐1 inhibition. N Engl J Med 377, 2500–2501. 29262275 10.1056/NEJMc1713444 PMC6549688

[40] Ka S , Lee S , Hong J , Cho Y , Sung J , Kim HN , Kim HL & Jung J (2017) HLAscan: genotyping of the HLA region using next‐generation sequencing data. BMC Bioinformatics 18, 258. 28499414 10.1186/s12859-017-1671-3 PMC5427585

[41] Bai Y , Wang D & Fury W (2018) PHLAT: inference of high‐resolution HLA types from RNA and whole exome sequencing. Methods Mol Biol 1802, 193–201. 29858810 10.1007/978-1-4939-8546-3_13

[42] Dilthey AT , Mentzer AJ , Carapito R , Cutland C , Cereb N , Madhi SA , Rhie A , Koren S , Bahram S , McVean G *et al*. (2019) HLA*LA‐HLA typing from linearly projected graph alignments. Bioinformatics 35, 4394–4396. 30942877 10.1093/bioinformatics/btz235 PMC6821427

[43] Newman AM , Liu CL , Green MR , Gentles AJ , Feng W , Xu Y , Hoang CD , Diehn M & Alizadeh AA (2015) Robust enumeration of cell subsets from tissue expression profiles. Nat Methods 12, 453–457. 25822800 10.1038/nmeth.3337 PMC4739640

