# Supplementary figures and images for: Benchmarking HLA genotyping and clarifying HLA impact on survival in tumor immunotherapy
Source: Mol Oncol. 2021 Jan 24;15(7):1764–82. doi: 10.1002/1878-0261.12895 (PMC8253103; doi:10.1002/1878-0261.12895)

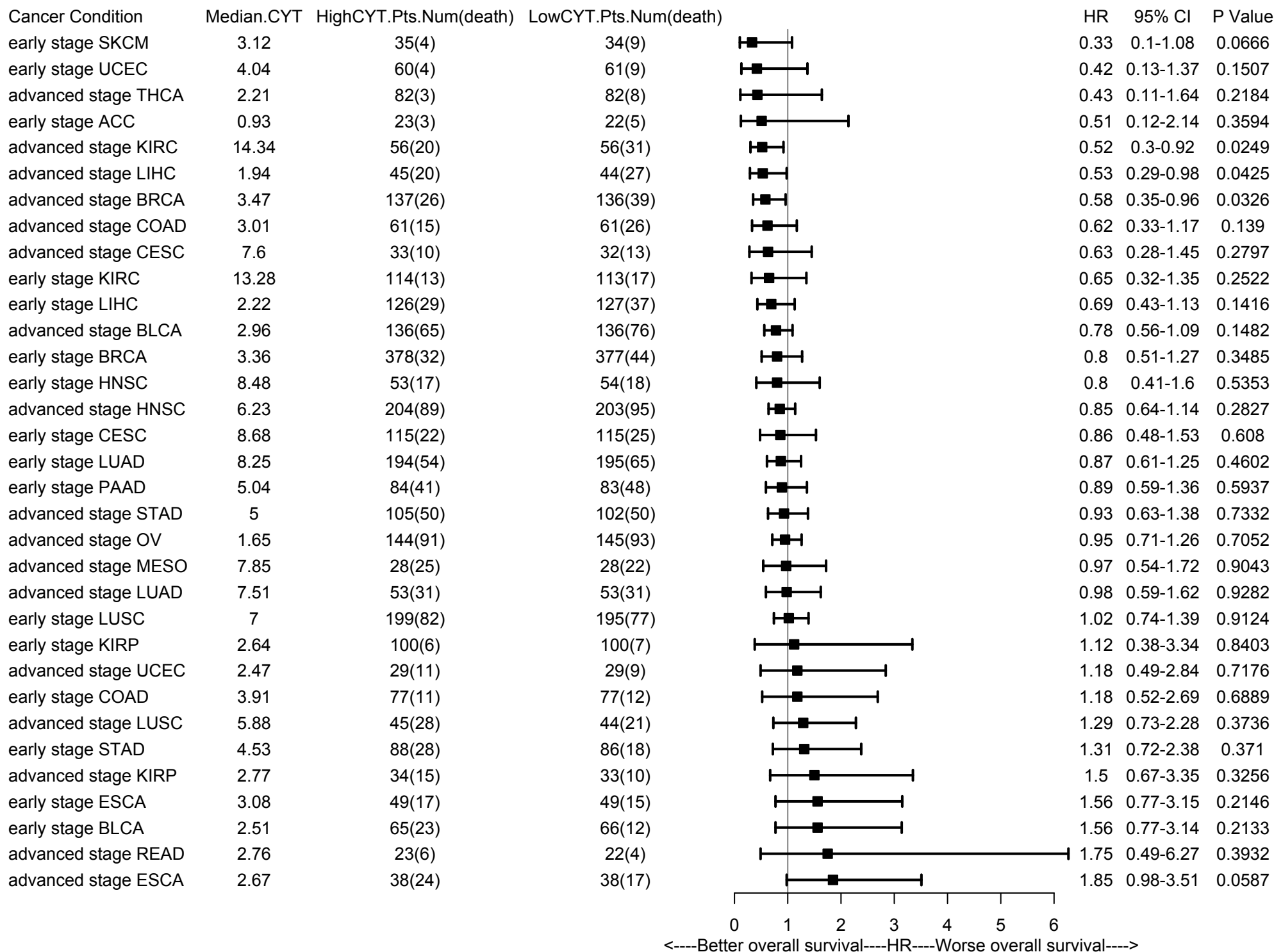

Supplement: Supplementary file 1 — Fig. S1. The survival impact of CYT in TCGA cancer conditions. [file MOL2-15-1764-s015.pdf]

A

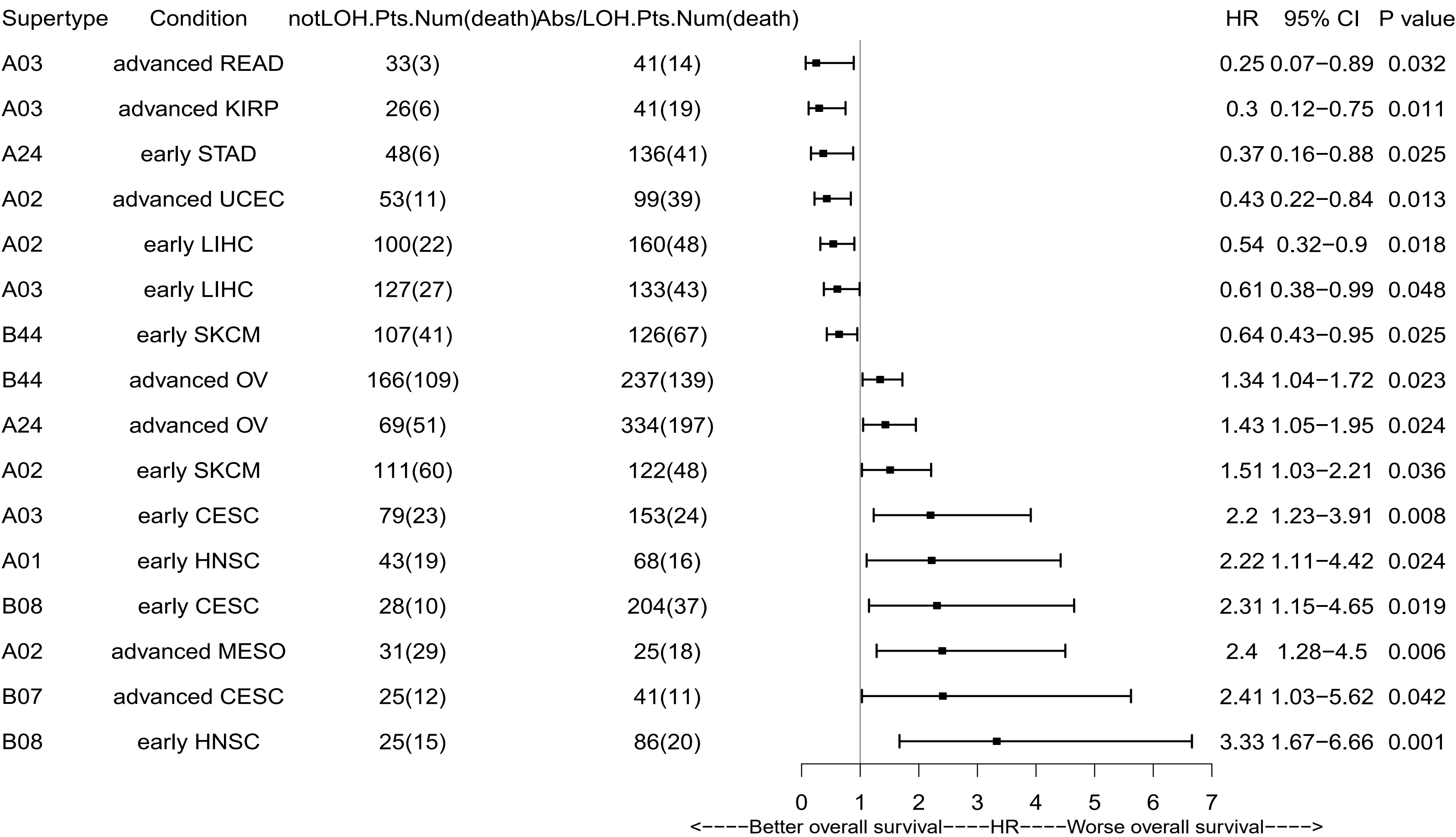

B

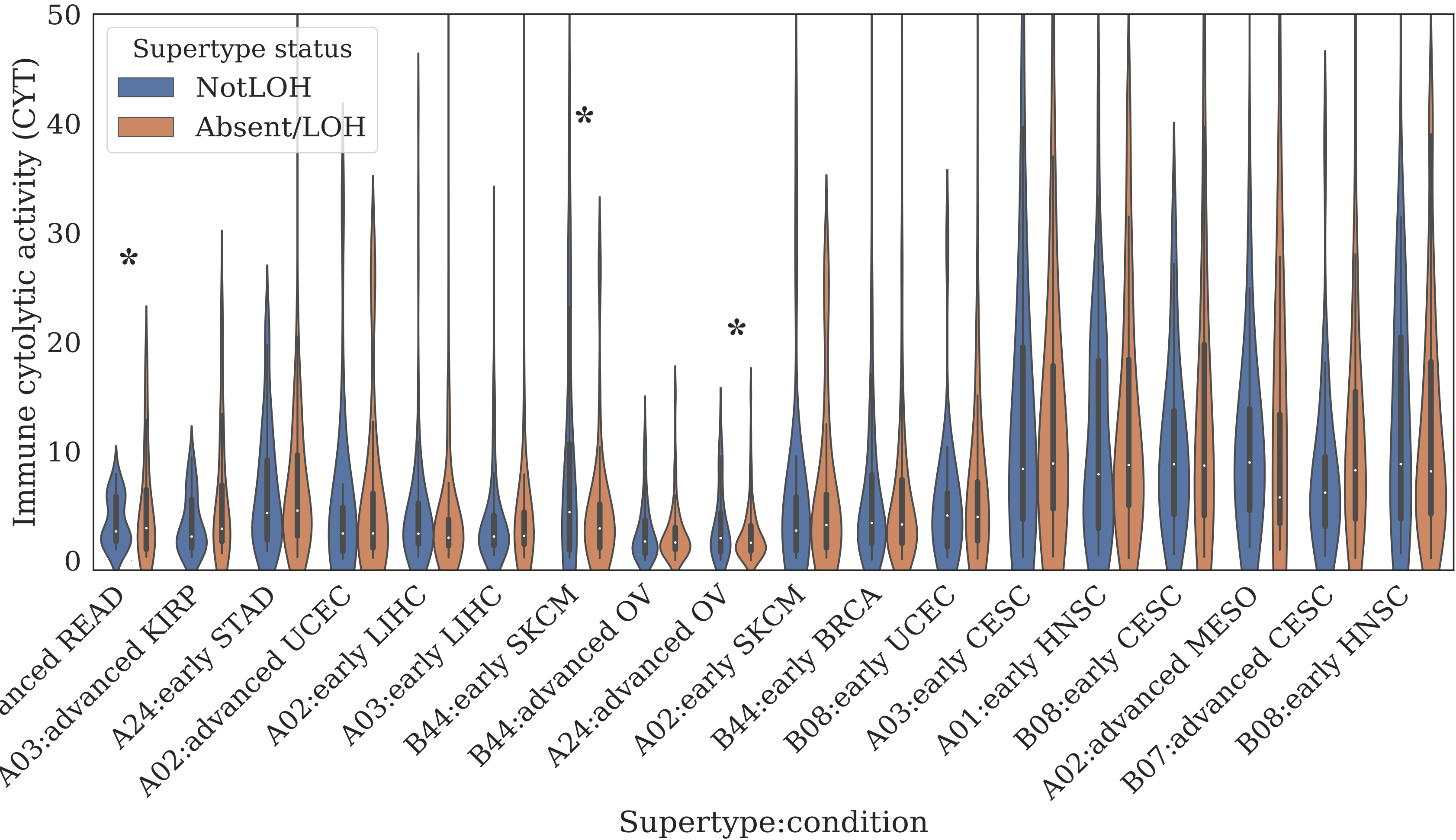

Supplement: Supplementary file 2 — Fig. S2. The survival impact of HLA alleles (with consideration of LOH status) in TCGA cancer conditions. [file MOL2-15-1764-s009.pdf]

A

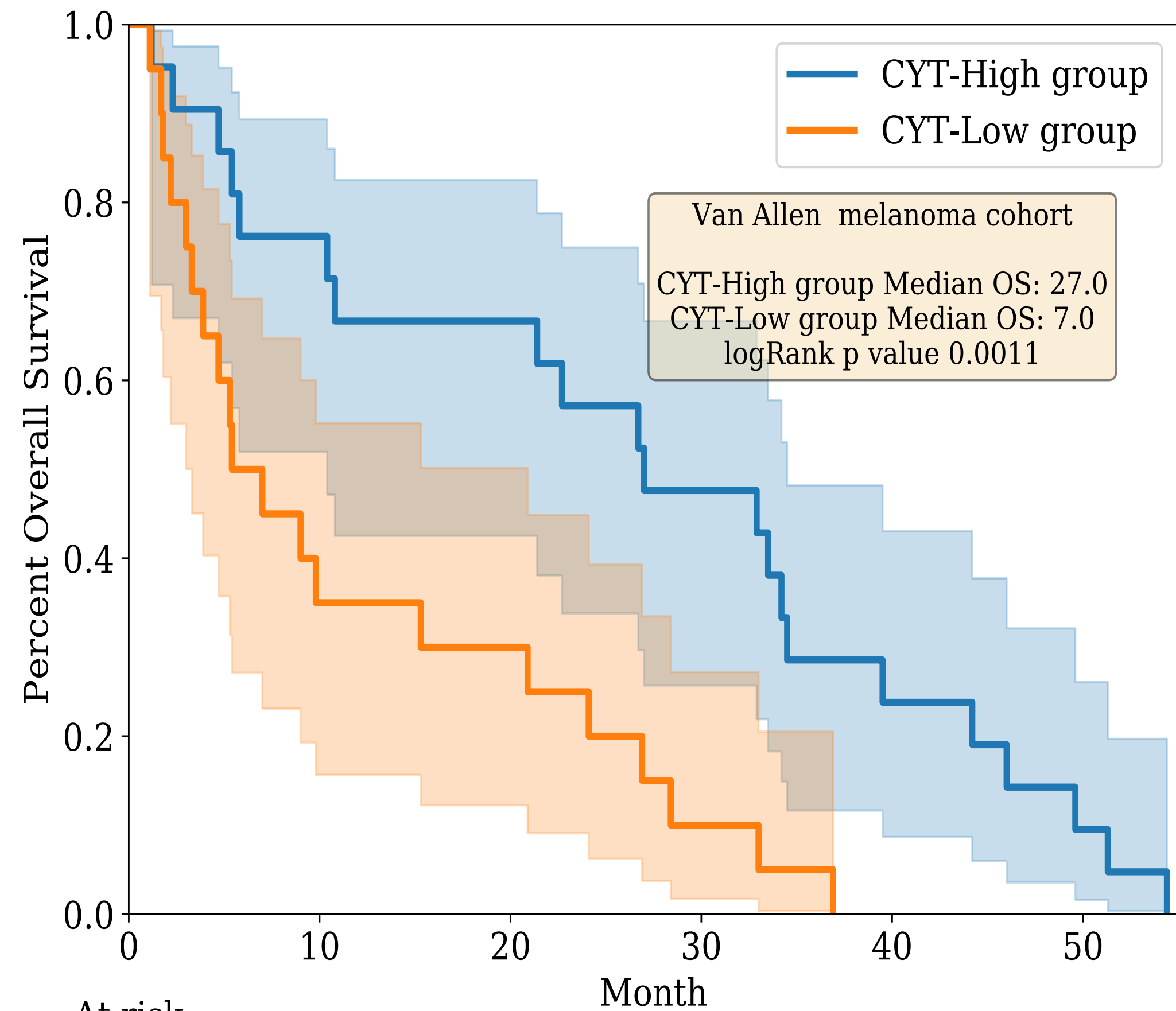

CYT-High group  
CYT-Low group

B

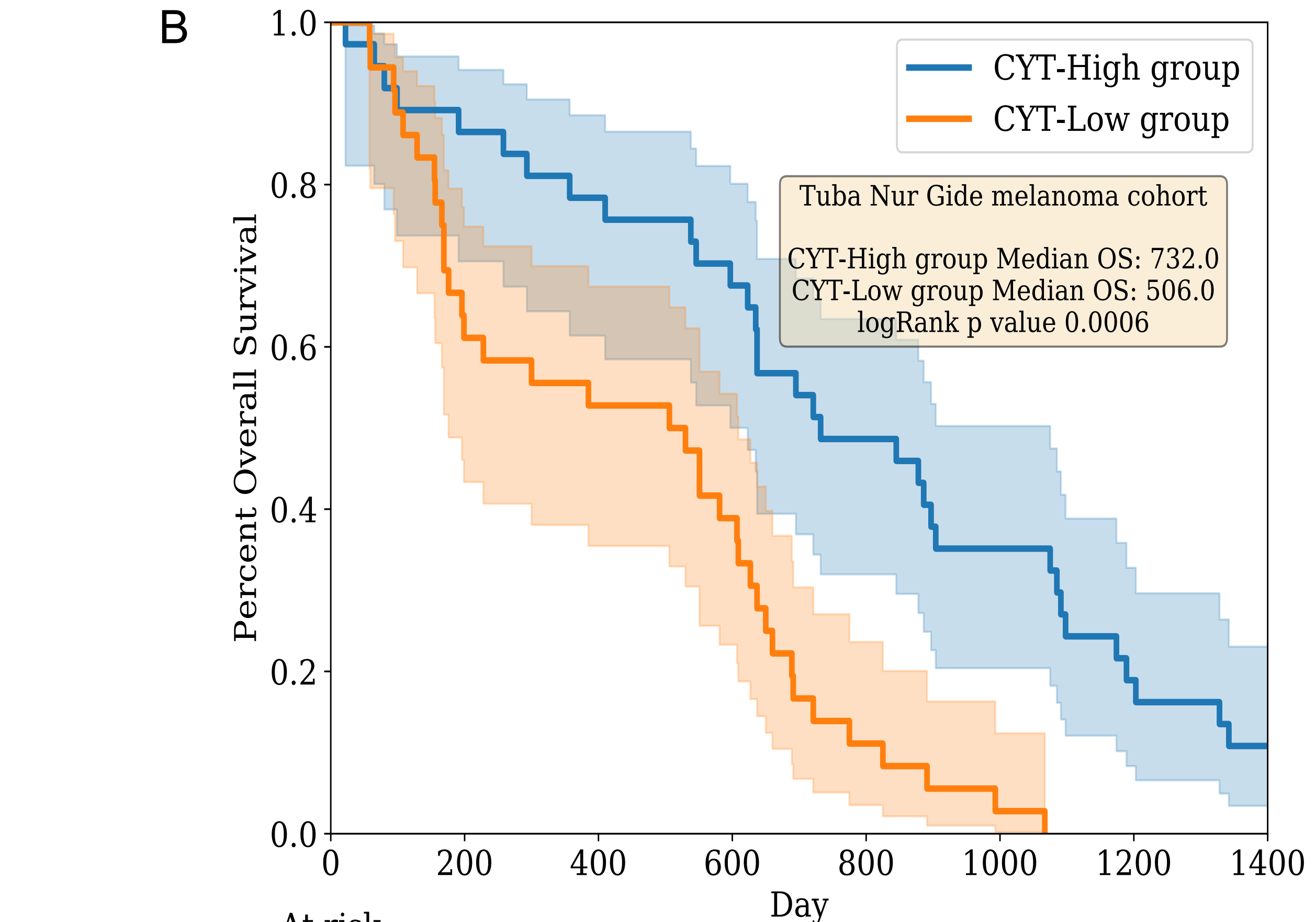

CYT-High group  
CYT-Low group

Supplement: Supplementary file 3 — Fig. S3. The survival impact of CYT in two ICB‐treated melanoma cohorts. [file MOL2-15-1764-s013.pdf]

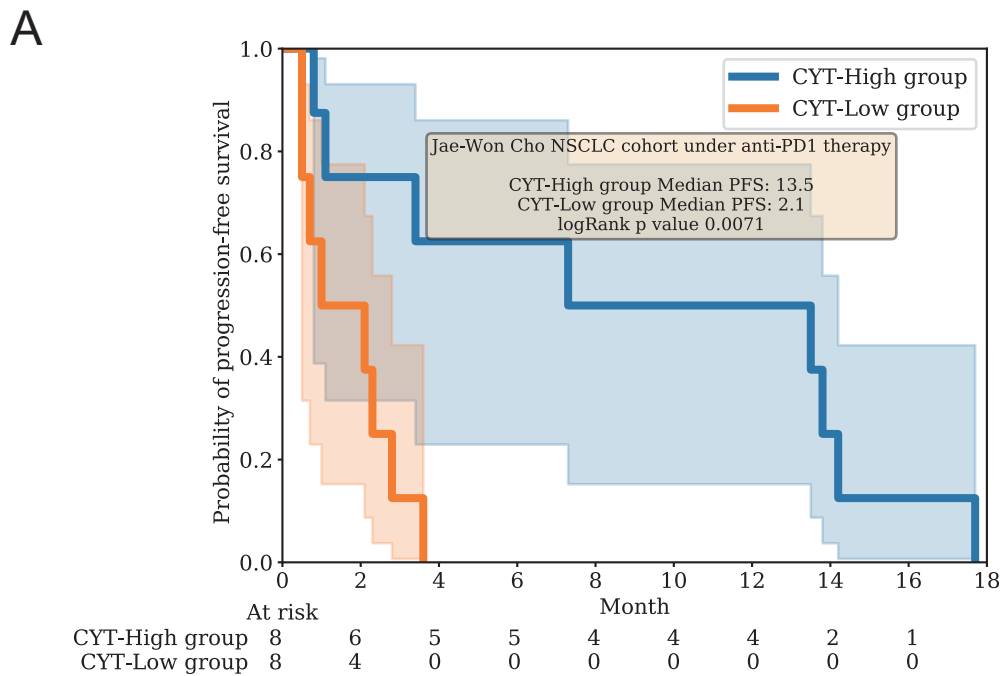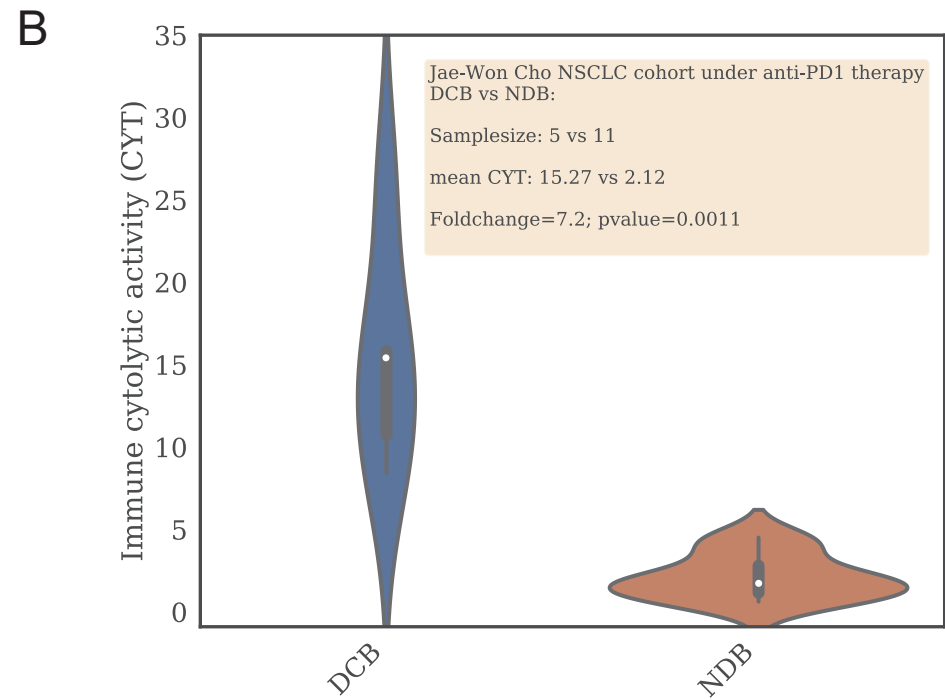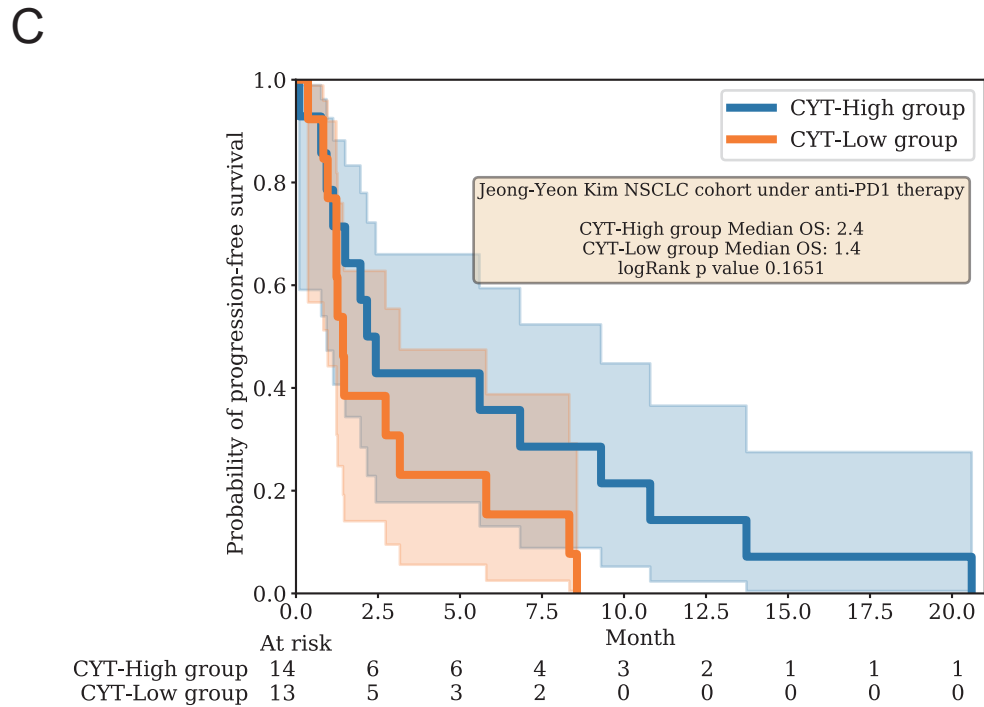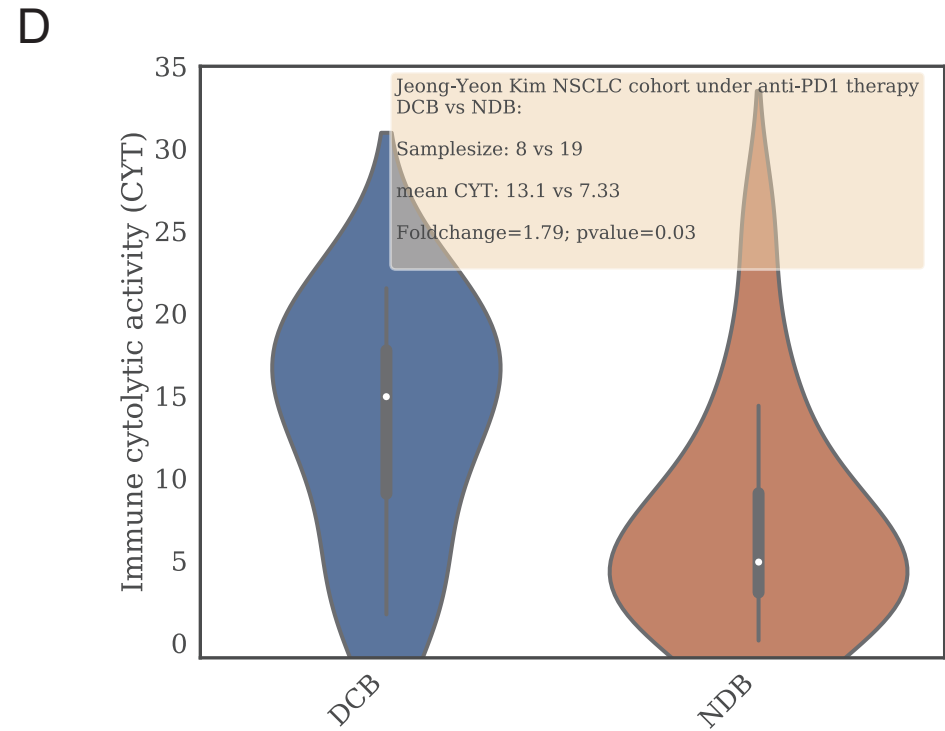

Supplement: Supplementary file 4 — Fig. S4. The survival impact of CYT in two ICB‐treated NSCLC cohorts. [file MOL2-15-1764-s003.pdf]

A

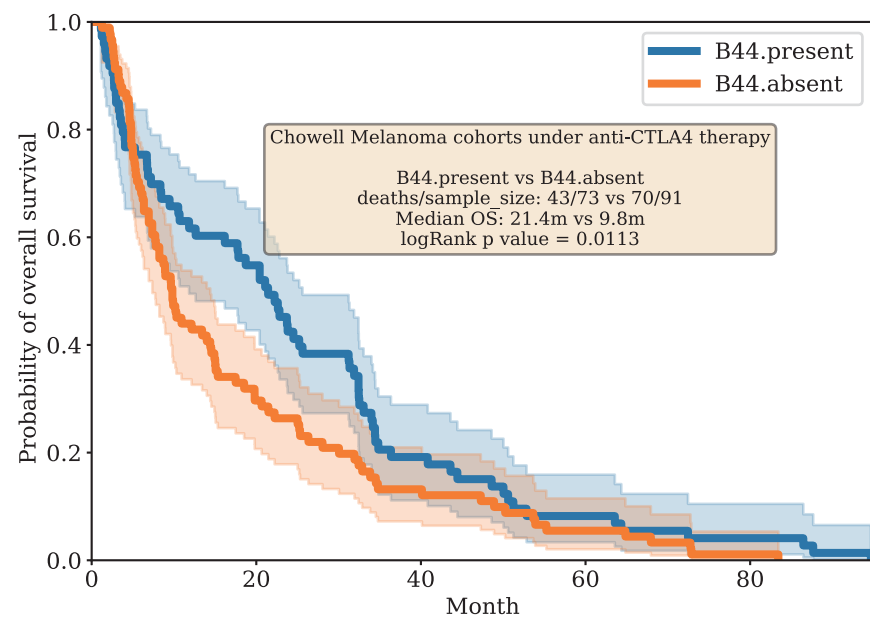

B

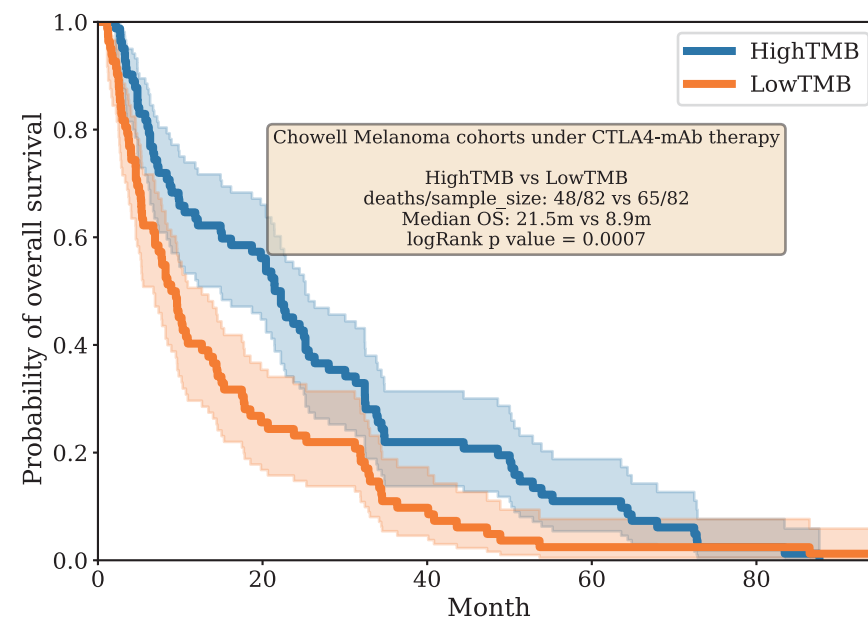

C

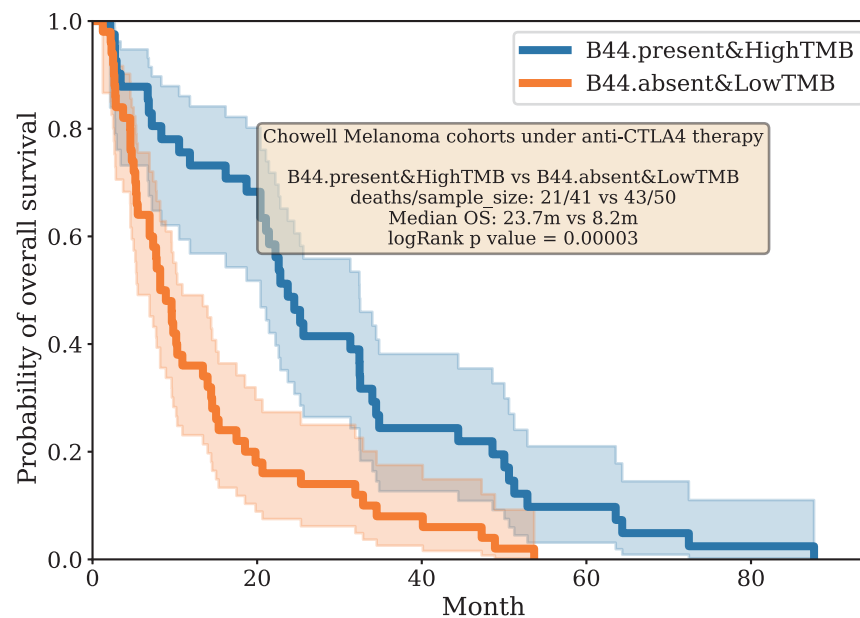

Supplement: Supplementary file 5 — Fig. S5. The survival curve in Chowell's ICB‐treated melanoma cohort. [file MOL2-15-1764-s002.pdf]

A

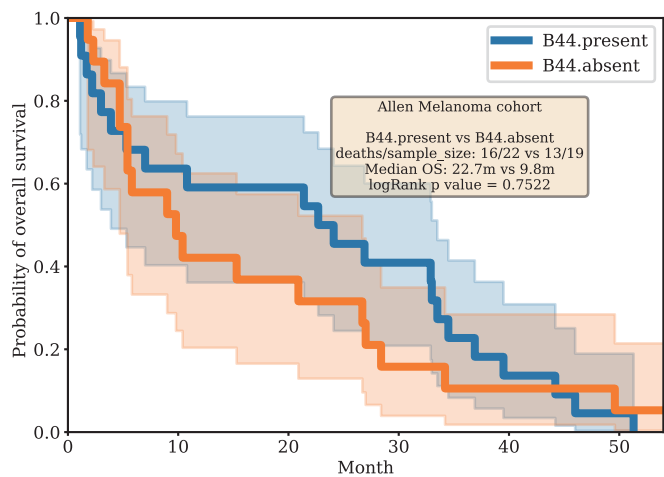

B

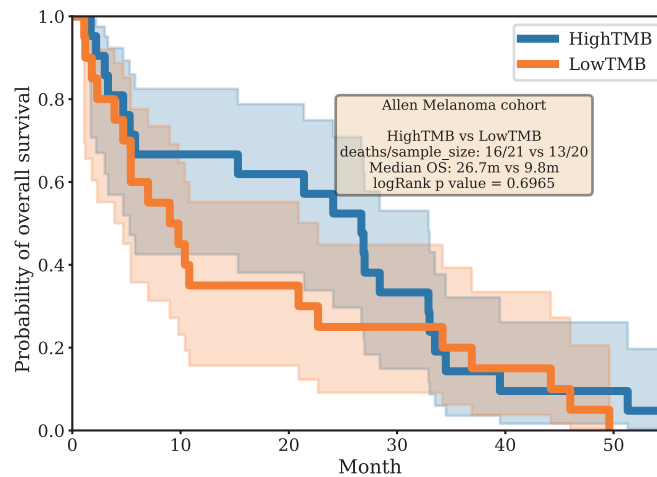

C

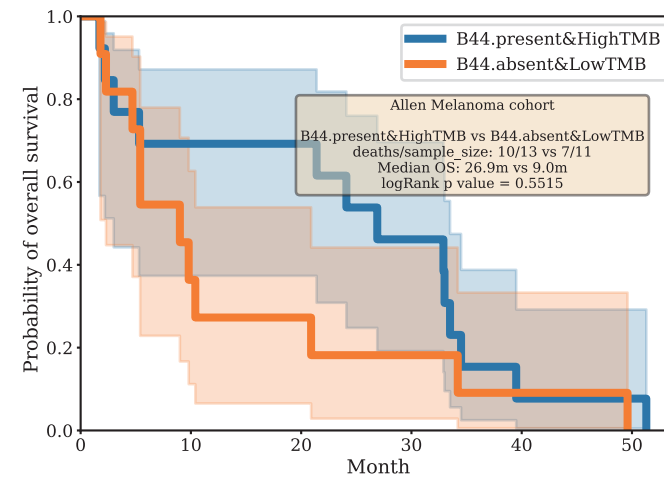

D

Corresponding CYT ↓

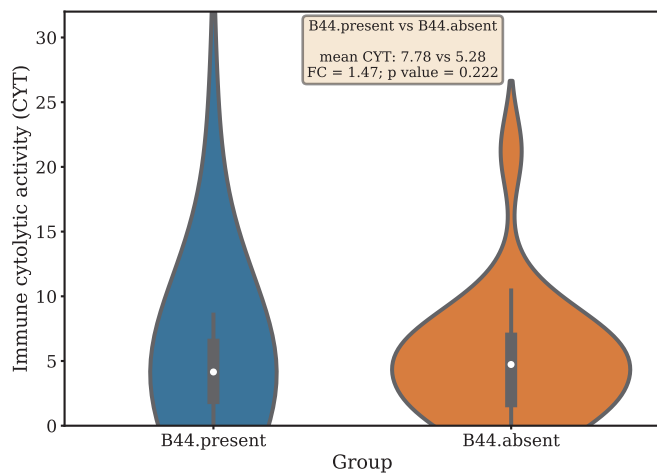

E

Corresponding CYT ↓

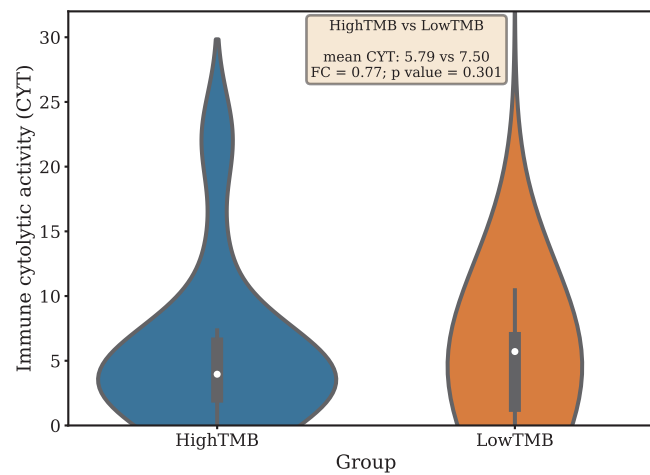

F

Corresponding CYT ↓

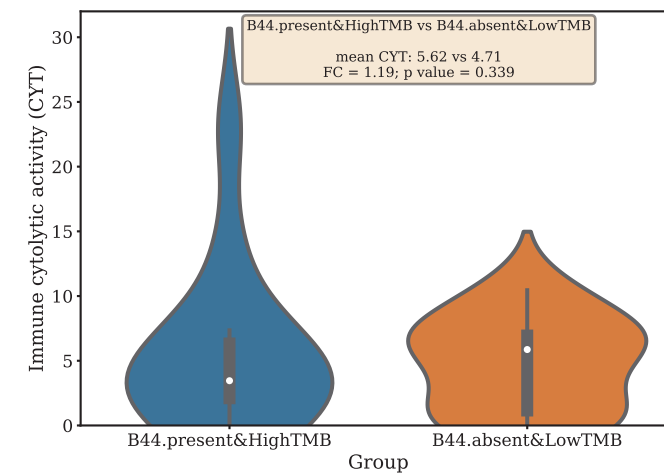

Supplement: Supplementary file 6 — Fig. S6. The survival plot and corresponding violin plot on CYT in Van Allen's melanoma cohort. [file MOL2-15-1764-s005.pdf]

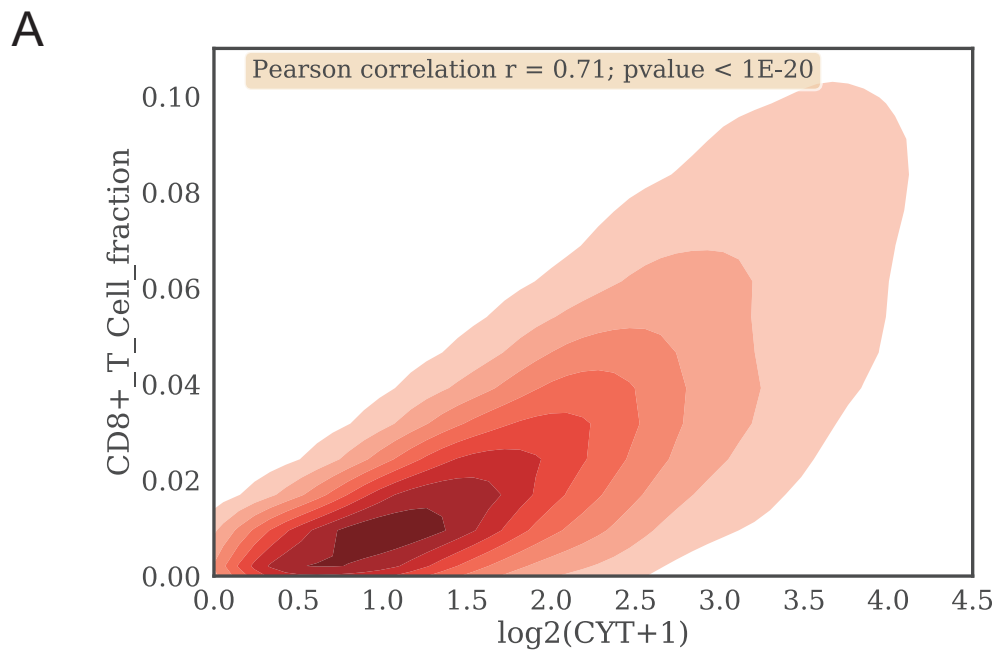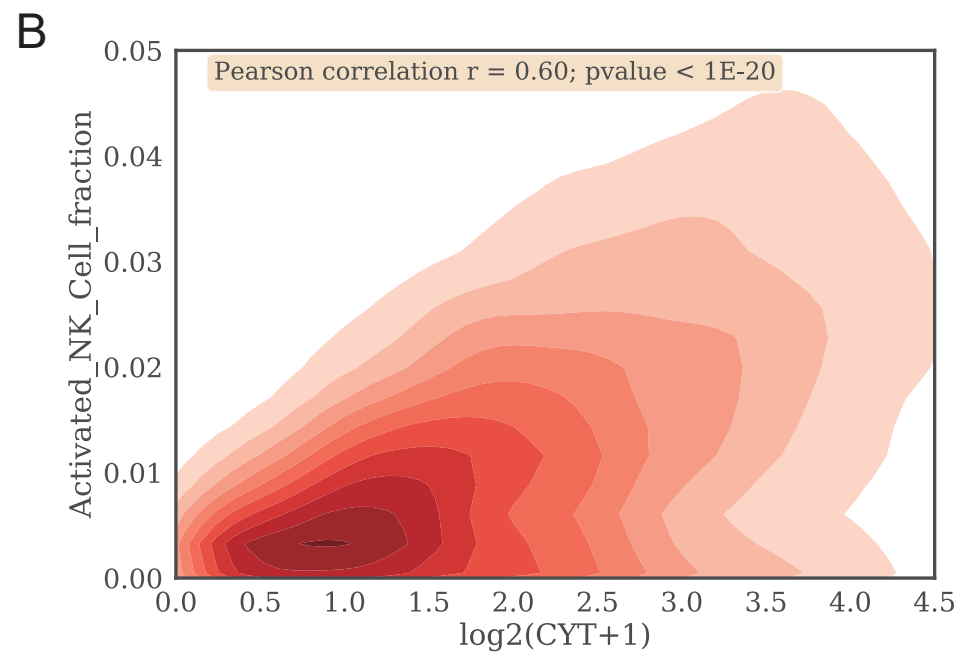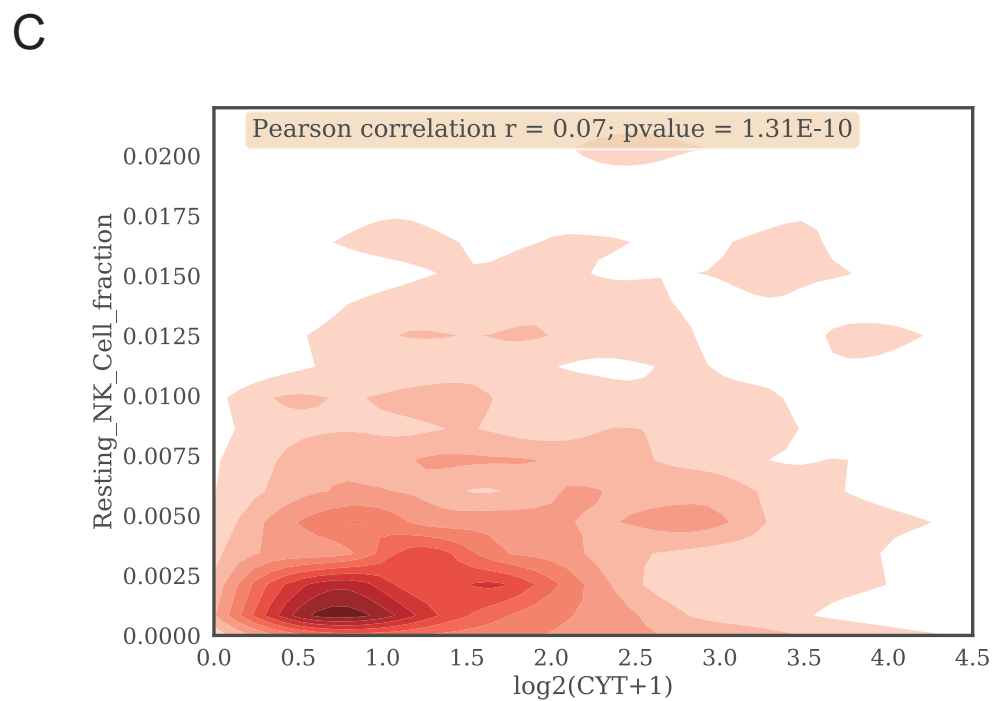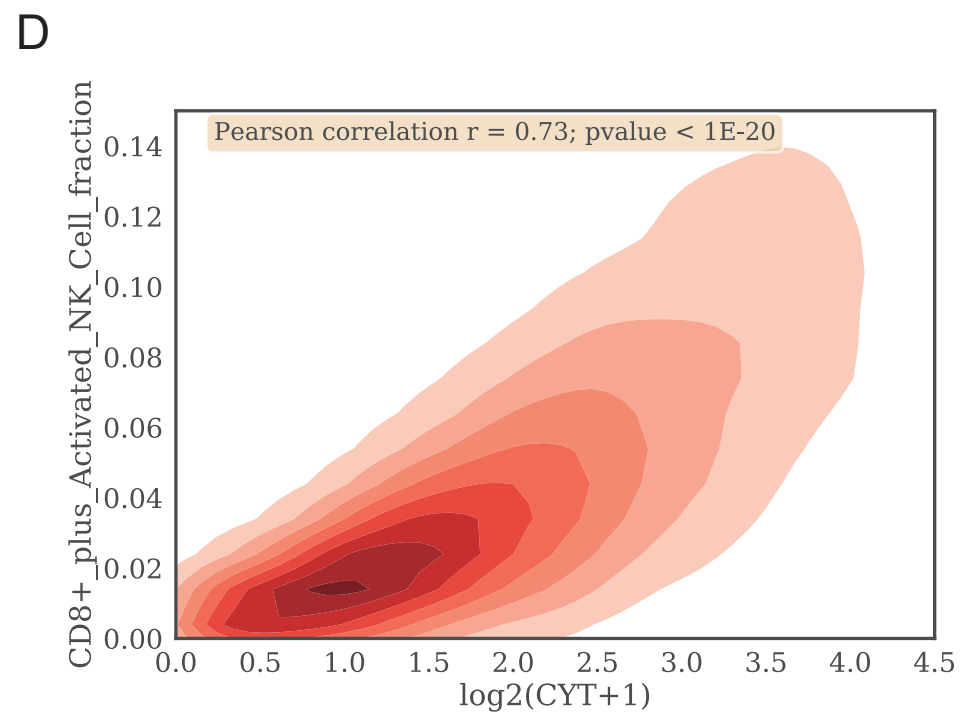

Supplement: Supplementary file 7 — Fig. S7. The correlation between CYT and cell fraction evaluated by CIBERSORT in absolute model. [file MOL2-15-1764-s014.pdf]
